# Human biomonitoring of mycotoxins: key challenges and future directions

**DOI:** 10.1007/s12550-025-00612-2

**Published:** 2025-12-23

**Authors:** Benedikt Cramer, Lia Visintin, Elias Maris, Michael Kuhn, Gisela H. Degen, Paul C. Turner, Hans-Ulrich Humpf, Sarah De Saeger

**Affiliations:** 1 Institute of Food Chemistry, University of Münster, Corrensstraße 45, D-48149 Münster, Germany; 2 Department of Bioanalysis, Centre of Excellence in Mycotoxicology and Public Health, Faculty of Pharmaceutical Sciences, Ghent University, Ghent, 9000 Belgium; 3 Department of Diagnostic Sciences, Doping Control Laboratory, Faculty of Medicine and Health Sciences, Ghent University, Ghent, 9000 Belgium; 4 Department of Microbiology and Immunology, Laboratory of Molecular Bacteriology, Rega Institute, KU Leuven, Leuven, 3000 Belgium; 5 Leibniz Research Centre for Working Environment and Human Factors (IfADo), Ardeystraße 67, D-44139 Dortmund, Germany; 6 Department of Global, Environmental and Occupational Health, School of Public Health, University of Maryland, College Park, MD USA; 7 Department of Biotechnology and Food Technology, Faculty of Science, University of Johannesburg, Doornfontein Campus, P.O. Box 17011, Johannesburg, South Africa

**Keywords:** Mycotoxins, Human biomonitoring, Analysis, Biomarker, Blood, Urine, Health risk, Exposure, Risk assessment

## Abstract

Mycotoxins pose a major health risk for humans in many parts of the world. For exposure assessment human biomonitoring (HBM) based on the analysis of serum, plasma, whole blood, urine or breast milk samples is a powerful approach which has been extensively used. However, there are several major challenges and pitfalls to be considered to achieve reliable and standardized data which can be used for risk assessment. These challenges include sampling (spot urine, 24 h urine, plasma, serum etc.) and analytical issues (missing reference standards, matrix effects, protein adducts, recovery, limit of detection, stability during storage, etc.). Further critical aspects needed for biomarker interpretation and human health risk evaluation are a lack of data for several mycotoxins such as (a) health-based guidance values (b) long-term biomarkers, (c) reliable food intake data (d) toxicokinetic parameters in humans, for example clearance and transfer rates to urine or milk and (e) HBM guidance values. In this review, these issues will be discussed based on recent HBM studies and recommendations for future studies as well as research needs will be derived.

**Graphical abstract:**

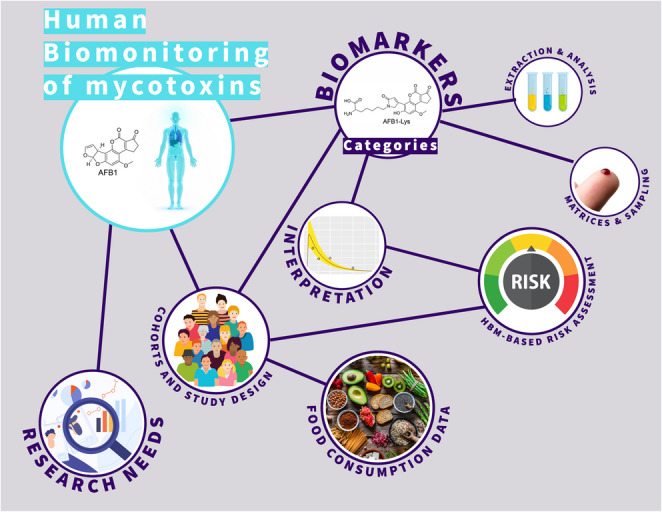

**Supplementary Information:**

The online version contains supplementary material available at 10.1007/s12550-025-00612-2.

## Introduction and objectives

### Mycotoxins and their occurrence

Mycotoxins are naturally occurring toxic compounds produced by certain fungi and present a major challenge to global food safety and public health. Toxigenic fungal species are usually able to produce more than one mycotoxin, and the same mycotoxin is often produced by different fungal species. To date, over 400 mycotoxins have been identified across a wide range of chemical classes and properties (Bräse et al. 2009). Fungal infection and consequent mycotoxin contamination can occur at different stages of crop or food production such as growth, cultivation, harvesting, transportation, processing, and storage. Fungal growth and mycotoxin production can be promoted at each stage by different physicochemical, and biological factors. Commonly contaminated crops are cereals, nuts and seeds, spices, and fruits. Mycotoxins can enter the human body via ingestion of contaminated plant-based food or by carryover via animal-derived food from livestock fed with contaminated feed (Al-Jaal et al. 2019; Arce-López et al. 2020). Furthermore, exposure can also occur via inhalation or dermal contact, e.g. by waste workers or farmers exposed to dust during harvesting or milling but also by indoor air inhalation (Al Hallak et al. 2023; Marcelloni et al. 2024). Mycotoxins induce a broad spectrum of acute and chronic toxic effects in both humans and animals (Sweeney 1998). The health outcomes vary by mycotoxin and depend on dose and duration of exposure, while the severity of symptoms may be modulated by differences in individual toxicokinetics and can manifest differently by age, health status and gender in some situations (Bennett and Klich 2003). Global estimates of the frequency of mycotoxin contamination in agricultural products are complex, but they have been estimated to affect between 25% and 80% of products (Eskola et al. 2020). Climate change is predicted to intensify public health risks, with expected changes related to occurrence of certain mycotoxins, levels and the frequency of exposures (Moretti et al. 2019; Casu et al. 2024). An additional concern is the overwhelming evidence that humans are exposed to multiple mycotoxins (Kyei et al. 2022; Nguyen et al. 2023), though the risks from such mixtures remain poorly examined.

Fungal prevalence and stress that lead to mycotoxin production have both crop specificities and climate specificities (Pitt et al. 2012), and those of highest public health concern are sometimes crudely placed into high risk or low risk of occurrence (Pitt et al. 2012) based on being produced in tropical (e.g. aflatoxins and fumonisins in sub-Saharan Africa or central America) or in more temperate regions (e.g. deoxynivalenol (DON), ochratoxin A (OTA) and zearalenone (ZEN) in Europe and North America). However, these regional classifications should not be interpreted as exclusive to those mycotoxins, and climate change is predicted to modify these geographic regions.

### Risk assessment based on external exposure assessment

Risk assessments of mycotoxins for humans and animals have been performed by the European Food Safety Authority (EFSA), the Joint FAO/WHO Expert Committee on Food Additives (JECFA), and national food safety authorities, highlighting health concerns at varying levels (EFSA 2012a, 2012b, 2014a; Riley et al. 2018). Traditionally, risk characterization is performed by correlating food consumption data with food contamination levels and comparing the population exposure with health-based guidance values (HBGV) if these are available. Alternatively, in case an HBGV could not be calculated due to uncertainty, or, in case the compound is genotoxic and carcinogenic, the point of departure (POD) is used. Derivation of the HBGV and POD is based on toxicity data from animal experiments and mode of action. When an HBGV is available, risk characterization is performed based on metrics such as the Hazard Quotient (HQ) or the Margin of Safety (IPCS 2009; More et al. 2019). For genotoxic and carcinogenic compounds or compounds for which data presents high uncertainty on their effects and no intake can be defined as safe, the Margin of Exposure (MoE) approach is used, as suggested by EFSA (Barlow et al. 2005). Performing risk assessment based on food contamination presents numerous challenges connected to the determination of occurrence data in food as well as food consumption data. The wide variety of food matrices that require analysis, along with the intrinsic heterogeneity in mycotoxin distribution within a batch and the presence of modified mycotoxins (Rychlik et al. 2014) which remain usually undetected in routine analysis, lead to a disproportionate analytical effort, with a high uncertainty on the estimation of ”average” contamination. Exposure assessment based on occurrence data may not always capture differences in bioaccessibility of mycotoxins from different food matrices that have been suggested from in vitro digestion studies (González-Arias et al. 2013; Rebellato et al. 2021; de Sá et al. 2024), though confirmatory human data are lacking. While most studies on mycotoxins are focused on dietary exposure, they often overlook exposure via inhalation and dermal absorption that have been reported for specific occupational categories (Marcelloni et al. 2024; Viegas et al. 2018). Another limitation of classical risk characterization based on food contamination data lies in the reliance on population-level food consumption data, which may fail to capture individual dietary patterns with sufficient resolution. This introduces an additional layer of uncertainty when characterizing the risk from external exposure estimates.

### The potential of human biomonitoring

Human biomonitoring (HBM) represents an alternative approach to assess individual human exposure overcoming the above-mentioned limitations. HBM captures the impact of all exposure sources and includes the influence of toxicokinetics and bioaccessibility. The potential of HBM extends far beyond the determination of the exposure to a single mycotoxin or mixtures of mycotoxins and allows various study designs to address specific research objectives (Choi et al. 2017). HBM study designs include, among others, (i) exposome studies (Wild 2005; Miller and Jones 2014; Gu et al. 2025; Hernandes et al. 2025), which focus on the comprehensive assessment of all exposures integrating multiple-omics technologies to understand cumulative risks; (ii) observational studies, which investigate exposure levels targeting specific populations to identify high-risk groups and evaluate interventions in developing countries where food surveillance systems are lacking or poorly implemented (Tesfamariam et al. 2022; Kyei et al. 2023; Bastos-Moreira et al. 2023; Phillips et al. 2025); (iii) toxicokinetic studies for the determination of the absorption, distribution, metabolism, and excretion of contaminants (Asam et al. 2013; Degen et al. 2018; Vidal et al. 2018a); (iv) case-control studies and longitudinal cohort studies, which are necessary to correlate exposure and health outcomes (Claeys et al. 2020); and (v) occupational studies to investigate the specific exposure in workplace environments (literature reviewed in Marcelloni et al. 2024).

### Limitations of human biomonitoring and objectives

HBM relies not only on validated analytical tools, but also on a clear understanding of the relationship between exposure to a toxin and concentration of that toxin (or metabolite) in a biological sample. Any toxin that is measured in a biological sample is referred to here as a bio-measure; however, where a demonstrated relationship is established between exposure and that bio-measure, the term biomarker is used. Bio-measures provide qualitative data indicative of exposure, whereas biomarkers provide both occurrence information and the ability to quantify that exposure. Further, this requires comprehensive toxicokinetic data to interpret biomarker levels and relate them to exposure and risk. Many biomarkers reflect only recent exposure; consequently, a major limitation is the lack of reliable biomarkers for assessing a long-term or cumulative exposure. Importantly, HBM data cannot reveal exposure routes or sources, as they represent an integration of exposure sources.

In this review, the challenges and opportunities associated with HBM of mycotoxins are explored, emphasizing practical solutions for advancing the field. All major mycotoxins in terms of toxicity and occurrence in various geographical regions will be covered. The availability of validated mycotoxin biomarkers and their analytical reference standards will first be discussed. Following this, different cohort study designs and cross-sectional studies will be presented, including their strengths, limitations, and key considerations for optimal planning. Sampling conditions and matrices, including alternatives to traditional blood withdrawal and methods for long-term exposure assessment, will also be examined. In addition, a review of the most common and promising analytical techniques in the field of mycotoxin HBM including their limitations will be provided. The discussion will then focus on biomarker interpretation and the role of HBM in the risk assessment of mycotoxins. This review will conclude with a summary on the state of the art and recommendations in form of bullet points as checklist on what is required in the future to fully benefit from the potential of mycotoxin HBM.

Besides the actual aspects covered in this review, the authors would like to refer scientists in the field to a selection of previous review papers on HBM of mycotoxins in general (Vidal et al. 2018b; Al-Jaal et al. 2019; Turner and Snyder 2021; Habschied et al. 2021) and to reviews covering HBM of specific mycotoxins such as aflatoxin B_1_ (AFB1) (Kensler and Eaton 2024), citrinin (CIT) (Ali and Degen 2019), DON (Schmied et al. 2023), ZEN (Mally et al. 2016) and OTA (Duarte et al. 2011; Malir et al. 2016).

## Biomarkers

Most mycotoxins undergo phase 1 and phase 2 metabolism. Consequently, a multitude of mycotoxin metabolites can theoretically be detected in human urine, blood, breast milk, and other bodily fluids. Many of these metabolites were initially identified in animal studies, and the resulting analytical methods and data were later applied to investigate human metabolism and exposure. However, for certain mycotoxins such as OTA, although several metabolites have been proposed or detected in vitro (Malir et al. 2016; Muñoz et al. 2017; see Sect. Ochratoxins), the fate of the parent compound remains only partially elucidated contributing to uncertainty in the interpretation of urinary HBM data based on the established biomarker OTA. The analysis of mycotoxin metabolites in human blood and urine primarily aims to provide information on exposure to the parent compound(s). Additionally, it may offer insights into the effective dose of a reactive metabolite, e.g. AFB1 epoxide, contribute to understanding the toxicity of mycotoxins, or help to investigate the impact of intervention measures (Warth et al. 2016a; Kensler and Eaton 2024).

**Fig. 1:**
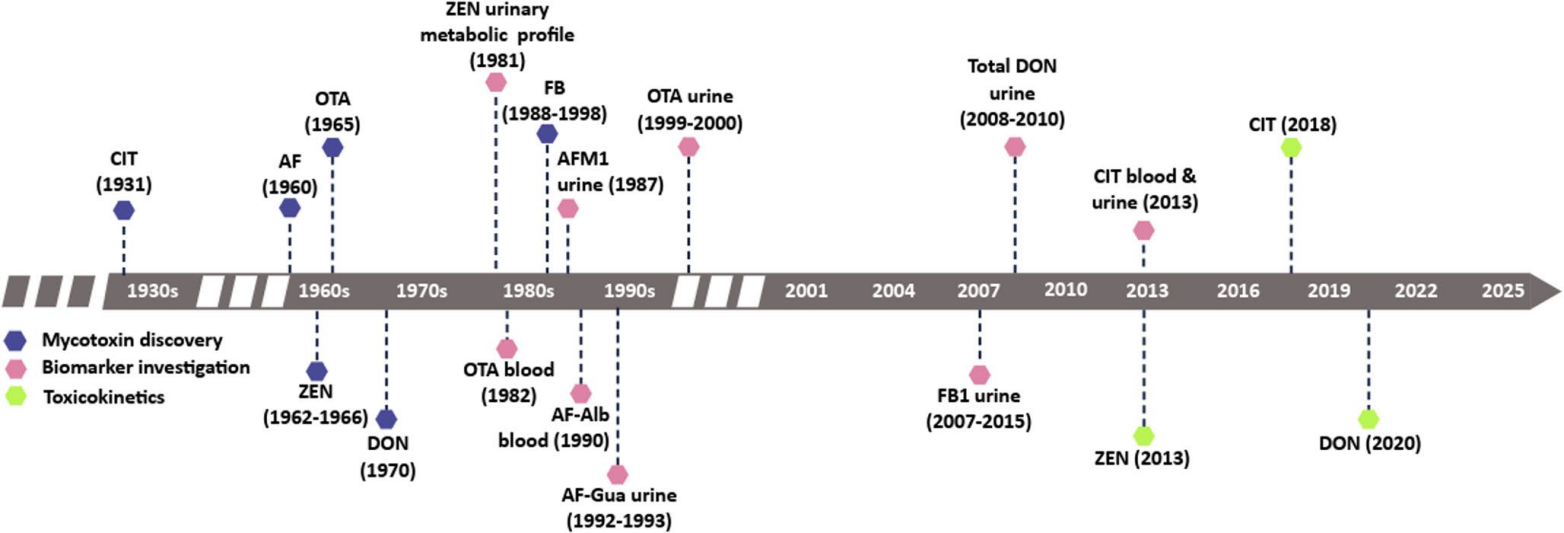
Timeline for discovery, biomarker investigations, and toxicokinetic studies for selected mycotoxins: citrinin (CIT), aflatoxins (AF), ochratoxin A (OTA), deoxynivalenol (DON), fumonisins (FB), and zearalenone (ZEN) as well as some main metabolites: aflatoxin M1 (AFM1), aflatoxin albumin (AF-Alb), and aflatoxin guanin (AF-Gua). It illustrates the often rather long period between discovery and further steps for developing human biomonitoring of a given mycotoxin

When assessing exposure, the most critical aspect is the correlation between bio-measure in body fluids and the intake of the parent compound. Only when that measure has been validated in human studies such that we can accurately rely on that bio-measure concentration to estimate exposure/ingestion of the mycotoxin we define this as a human biomarker of exposure. A bio-measure alone typically is indicative of some exposure, whereas the biomarker quantifies that exposure, albeit typically with some margin of error in that estimate (Turner and Snyder 2021). The population used in establishing this data may be important, for example age or study size may skew or misrepresent interpretation (Santonen et al. 2023). For some compounds, only toxicokinetic data obtained in animals are available. Although often collected under more controlled conditions compared to human studies, these data are not always relevant to humans. An example is the formation of DON-15-glucuronide (DON-15-GlcA) as the primary human metabolite of DON, which is not observed in most animal species (Maul et al. 2012). Moreover, understanding differences in sample preparation, such as treatment with glucuronidase or sulfatase, as well as the analytical instrumentation applied are critical. OTA is an example where renal clearance in humans was determined without glucuronidase treatment, although the presence of glucuronides has been suggested (Muñoz et al. 2010). Ultimately, establishing a link between mycotoxin biomarkers and biological effects in humans and/or animals is necessary for biomonitoring-based risk assessment. This aspect is discussed in detail in Sect. Recent trends and challenges in interpretation of human biomonitoring data. Figure 1 summarizes the timeline for discovery, biomarker investigations, and toxicokinetic studies for selected mycotoxins.

The National Research Council (2006) outlined a classification scheme for biological measurements which we have adopted and adapted to the specific requirements of HBM of mycotoxins (Table 1). In the field of mycotoxins, the identification and verification of biomarkers for various compounds is far from complete, and several mycotoxins and their metabolites are included in analytical methods only as bio-measures and putative biomarkers. Some of these compounds have not yet been detected in human biological fluids, while others have been detected by only one or two laboratories. To take this into account, categories 0–3 comprise bio-measures or not-yet-biomarker compounds, 4 covers biomarkers for which no correlation with a specific adverse effect is demonstrated, and categories 5–7 represent biomarkers that essentially allow at least preliminary risk assessment. Based on these categories, mycotoxin biomarkers were classified accordingly in the following sections. The chemical structures of all presented mycotoxins and metabolites can be found in the Supplementary Information.

**Table 1:** Categorization of mycotoxins and metabolites used in this publication (adapted from National Research Council (2006)

Category	Description
0	Compound implemented into analytical methods but not detected in the sample matrix. References report non-detects.
1	Compound detected with less than three different analytical methods. References report detects and non-detects.
2	Compound detected with three or more different analytical methods. No known relationship between analyte concentration and external dose. References report first and ongoing detects.
3	Compound detected with three or more different analytical methods. A relationship to external dose in animals exists. References report first and ongoing detects.
4	Compound detected with three or more different analytical methods. A relationship to external dose in humans exists but not to a biological effect. References report first and ongoing detects.
5	Compound detected with three or more different analytical methods. A relationship between the biomarker concentration and a biological effect in animals exists. References report first and ongoing detects.
6	Compound detected with three or more different analytical methods. A relationship between the biomarker concentration and external dose in humans exists. Relationship between biomarker concentration and biologic effect in animals exists. References report first and ongoing detects.
7	Compound detected with three or more different analytical methods. A relationship between the biomarker concentration, external dose, and biologic effect in humans exists.

### Aflatoxins

The four major aflatoxins produced by *Aspergillus* fungi are AFB1, aflatoxin B_2_ (AFB2), aflatoxin G_1_ (AFG1), and aflatoxin G_2_ (AFG2). Additionally, sterigmatocystin (STG), a toxic biosynthetic precursor of aflatoxins, can be found in infested materials. Aflatoxin M_1_ (AFM1), a metabolite of AFB1, can be detected in human breastmilk and urine, and the milk of other lactating mammals. AFB1 is the most toxic and carcinogenic aflatoxin and is often regarded as the lead compound for aflatoxin contamination of plant-based materials. Its metabolism has been extensively studied and reviewed (Kensler et al. 2011; Kensler and Eaton 2024), and its metabolites have been investigated for their suitability as biomarkers of exposure or effect in urine and blood. For urine, a correlation between AFM1 and aflatoxin B_1_-guanine (AFB1-Gua) excretion and AFB1 exposure has been demonstrated, with 0.1–6.1% (mean 1.49%) of AFB1 being excreted as AFM1 and 0.2% as AFB1-Gua in urine (Zhu et al. 1987; Groopman et al. 1993). No useful correlation for the parent compound AFB1 in urine, nor the aflatoxin P_1_ (AFP1) metabolite, with AFB1 exposure has been observed (Groopman et al. 1993). Among the other aflatoxins, the metabolism of AFG1 has been investigated in more detail, and the formation and excretion of aflatoxin M_2_ (AFM2) in human urine has been demonstrated. The compounds listed in Table 2 are mostly analysed in urine since the beginning of the 1980s. In recent UHPLC-MS-based HBM methods for urine, AFM1 AFB1, AFB2, AFG1, AFG2, AFM2, AFP1, and aflatoxin Q_1_ (AFQ1), are included, alongside many other mycotoxins and mycotoxin metabolites, to obtain a broader overview of their occurrence with less requirements for sample preparation (see Sect. Matrices, sampling, clean-up and mass spectrometric analysis) (Franco et al. 2019; De Ruyck et al. 2020). AFB1-Gua and AFB_1_-*N*-acetyl-cysteine (AFB1-NAC) are not part of recent HBM methods, most likely reflective of the lack of available reference standards. Aflatoxicol (AFL), another metabolite of AFB1, has also received little attention and was only part of older aflatoxin-specific methods, despite commercial availability.

For the analysis of aflatoxin exposure in hematic matrices, the detection of aflatoxin covalently bound to albumin (AF-Alb) via a lysine residue is the method of choice and the only validated biomarker for this fluid, with 1.4–2.3% of ingested AFB1 being converted to this compound (Gan et al. 1988). The AFB1-Lys adduct has to be liberated from albumin, where the ε-amino group of lysine reacts with activated AFB1 (reviewed by Kensler et al. 2011). This is achieved in serum and plasma samples by enzymatic digestion using a mixture of proteolytic enzymes (Pronase E), followed by sample clean-up, chromatographic separation and detection (for a recent method see Phillips et al. 2025). Identification of AFB1-Lys without prior enzymatic hydrolysis has also been reported (De Ruyck et al. 2020), which is rather unexpected, and additional confirmatory analyses are recommended. The AFB1-Lys standard is not commercially available, and a recently published method, reporting a straightforward synthesis, appears to generate primarily an isomer of the natural form, which must be considered when applying this standard (Renaud et al. 2022a, b). An AFG1-lysine adduct has been proposed to be formed but has not yet been detected in human samples (Sabbioni and Wild 1990), though a standard was recently produced (Renaud et al. 2022b).

As shown in Table 2, analyses of other aflatoxins in hematic matrices by UHPLC have also been performed since the early 1980s, but with mostly low abundance rates, except for studies where exposure to high levels of aflatoxins were recorded. In a recent publication, occasional detections for AFB2 were reported (De Ruyck et al. 2020).

In a study with vervet monkeys, approximately 15% of ingested STG was excreted within 24 h as glucuronide via urine (Steyn and Thiel 1976). In this study, only glucuronidated STG was observed using thin-layer chromatography (TLC) and radiolabelling. Additional in vitro studies indicate the formation of hydroxylated derivatives of STG, as well as *N*-acetyl-cysteine adducts of formed hydroxyquinones in humans (Pfeiffer et al. 2014). The first report on the presence of STG in human urine and blood was published by Cao et al. (2018) after glucuronidase treatment of urine and plasma samples. Since then, it has been detected without this pretreatment by different groups (De Ruyck et al. 2020; Tesfamariam et al. 2022; Ning et al. 2024). Currently, no correlation between STG levels in blood or urine and exposure has been demonstrated for humans and more research is needed to fully understand the human in vivo metabolism of STG.

**Table 2:** Aflatoxins and metabolites and their application for mycotoxin exposure assessment (Categories (Cat) according to Table 1, n/a: not applicable)

	Urine		Blood/serum/plasma		Comment
	Cat	Reference	Cat	Reference	
Aflatoxin B_1_ (AFB1)	3	Nelson et al. 1980^1^ Groopman et al. 1993^2^ De Ruyck et al. 2020^3^	3	Nelson et al. 1980^1^ Ritieni 2010^3^ De Ruyck et al. 2020^3^	No useful correlation between urinary AFB1 and exposure to AFB1 (Groopman et al. 1993).
Aflatoxin B_1_-lysine (AFB1-Lys)	0*	De Ruyck et al. 2020^4^	7*	Sabbioni 1990^1,2^ McMillan et al. 2018^3^ Philips et al. 2025^3^	After enzymatic release from protein. See Table 11.
Aflatoxin B_1_-gua (AFB1-Gua)	7*	Autrup et al. 1983^1^ Groopman et al. 1993^2^ Egner et al. 2006^3^ Njumbe Ediage et al. 2012^3^	n/a		No part of recently published quantitative methods, biomarker of effect. See Table 11.
Aflatoxin B_2_ (AFB2)	2	Jonsyn 1999^1^ De Ruyck et al. 2020^3^ Kyei et al. 2022^3^	2	Nelson et al. 1980^1^ Tesfamariam et al. 2022^3^	
Aflatoxin G_1_ (AFG1)	2	Qian et al. 1994^1^ Jonsyn et al. 1999^3^ De Ruyck et al. 2020^3^	1	Nelson et al. 1980^1^ Cao et al. 2018^3^ De Ruyck et al. 2020^3^	
Aflatoxin G_1_-lysine (AFG1-Lys)	n/a		1*	Sabbioni and Wild 1991	Not yet detected in humans but a potential explanation for elevated ELISA results (Sabbioni and Wild 1991).
Aflatoxin G_2_ (AFG2)	1	Jonsyn 1999^1^ De Ruyck et al. 2020^3^	2	Nelson et al. 1980^1^ Jonsyn 1999^3^ De Ruyck et al. 2020^3^	
Aflatoxin M_1_ (AFM1)	7	Campbell et al. 1970^1^ Groopman et al. 1985^2^ Solfrizzo et al. 2011^3^	2	Nelson et al. 1980^1^ Jonsyn 1999^3^ De Ruyck et al. 2020^3^	See Table 11.
Aflatoxin M_2_ (AFM2)	1	Jonsyn 1999^3^ Fan et al. 2019^4^	1	Fan et al. 2019^4^ Ning et al. 2024^4^	
Aflatoxin P_1_ (AFP1)	2	Groopman et al. 1985^1^ Franco et al. 2019^3^	n/a		No useful correlation between urinary AFP1 and exposure to AFB1 (Groopman et al. 1993).
Aflatoxin Q_1_ (AFQ1)	2	Qian et al. 1994^1^ Franco et al. 2019^3^ Braun et al. 2022^3^	0	De Ruyck et al. 2020^4^	
Aflatoxicol (AFL)	1	Jonsyn 1999^1^	1	Hendrickse et al. 1982^1^ Jonsyn 1999^3^	
Aflatoxin B_1_-mercapturic acid AFB1-NAC)	4*	Wang et al. 1999^1^	n/a		Not part of recently published quantitative methods.
Sterigmatocystin (STG)	1	Cao et al. 2018^1^ De Ruyck et al. 2020^3^	2	Cao et al. 2018^1^ De Ruyck et al. 2020^3^ Tesfamariam et al. 2022^3^	Analysis with and without glucuronidase.

*No commercially available standard. ^1^First report on the occurrence in humans. ^2^Report on quantitative relationship with exposure. ^3^Example of a recent method where the compound was detected. ^4^Example of a recent method where the compound was not detected

### Ochratoxins

HBM of OTA began with the analysis of human serum as part of the search for the causative agent of Balkan endemic nephropathy (Hult et al. 1982). Since then, numerous studies have analysed this compound in blood across many countries (Malir et al. 2016). Currently, published methods report over 98% of values above the limit of quantification (LOQ), which makes the dataset on OTA in blood probably the most comprehensive one available for mycotoxins (Jaus et al. 2022). Besides OTA, the occurrence of OTα and OTB in serum has been occasionally reported (Muñoz et al. 2010; Ning et al. 2024). Both compounds co-occur with OTA in food, with OTα also being reported as a metabolite of the human intestinal microbiota (Camel et al. 2012). 2’R-OTA, a thermal degradation product of OTA, is also frequently found in blood samples (Cramer et al. 2015; Jaus et al. 2022). OTA and its metabolites, along with their categorization, are listed in Table 3.

Approximately 2.5% of ingested OTA is excreted unchanged in urine within 24 h, as determined by radiometry and HPLC measurements (Studer-Rohr et al. 2000). A good correlation between OTA levels in urine and exposure has been demonstrated, making urinary OTA a suitable biomarker of exposure (Gilbert et al. 2001; Muñoz et al. 2014). However, the study by Studer-Rohr et al. (2000) additionally revealed that only 44–52% of the radioactivity excreted in urine came from unmodified OTA, clearly indicating the formation of phase 1 or phase 2 metabolites. Despite this, a clear and confirmed assignment of the remaining radioactivity to specific metabolites is still lacking. The formation of OTα may explain partially the fate of OTA, but the chemical structures of glucuronides of OTA and OTα, as well as hydroxylated derivatives of OTA, remain unconfirmed and are proposed solely based on mass spectrometric data (Han et al. 2013). Nevertheless, comparative analysis of urine samples with and without glucuronidase treatment supports the importance of glucuronides in OTA metabolism and elimination (Muñoz et al. 2010). The formation of conjugates with glutathione has also been described, although this has not been confirmed in additional studies (Sueck et al. 2020; Dekant et al. 2021).

**Table 3:** Ochratoxins, citrinin (CIT) and metabolites and their application for mycotoxin exposure assessment (Categories (Cat) according to Table 1, n/a: not applicable)

	Urine		Blood/serum/plasma		Comment
	Cat	Reference	Cat	Reference	
Citrinin (CIT)	6	Njumbe Ediage et al. 2012^1^ Blaszkewicz et al. 2013^3^ Heyndrickx et al. 2015^3^	3	Blaszkewicz et al. 2013^1^ De Ruyck et al. 2020^3^ del Mar Delgado-Povedano 2025^3^	See Table 11.
Dihydrocitrinone (DH-CIT)	6	Blaszkewicz et al. 2013^1^ Ali et al. 2015^3^ Heyndrickx et al. 2015^3^	0	Osteresch et al. 2016^4^	Main metabolite of CIT, often higher incidence in urine. See Table 11.
Ochratoxin A (OTA)	6	Castegnaro et al. 1990^1^ Gilbert et al. 2001^2^ Muñoz et al. 2010^3^ Gerding et al. 2015^3^	6	Hult et al. 1982^1^ Studer-Rohr et al. 2000^2^ Gilbert et al. 2001^2^ Cramer et al. 2015^3^	Main biomarker of OTA exposure in urine and blood. See Table 11.
Ochratoxin α (OTα)	2	Muñoz et al. 2010^1^ Ali et al. 2017b^3^ De Ruyck et al. 2020^3^	2	Muñoz et al. 2010^1^ Ali et al. 2017b^3^ De Ruyck et al. 2020^3^	Intestinal metabolite, prone to misidentification.
Ochratoxin B (OTB)	1	Jonsyn-Ellis 2001^1^ Ringot et al. 2006^3^	1	Ning et al. 2024^3^	Relevance as OTA metabolite questionable.
OH-metabolites of OTA (OH-OTA)	1	Jonsyn-Ellis 2001^1^ Njumbe Ediage 2012^3^	0	Osteresch et al. 2017^4^	No recent detects.
OTA-glucuronides (OTA-GlcA)	0	Pena et al. 2006^1^ Muñoz et al. 2017^3^	n/a		So far only indirect detection successful.
Otα-glucuronides (OTα-GlcA)	0	Muñoz et al. 2010^1^	0	Muñoz et al. 2010^1^	So far only indirect detection successful.
OTB-mercapturic acid (OTB-NAC)	1	Sueck et al. 2020^1^ Dekant et al. 2021^4^	n/a		In humans only detected in a single laboratory.
2’R-Ochratoxin A (2’R-OTA)	1	Viegas et al. 2018^1^ Schmidt et al. 2021b^4^	4	Cramer et al. 2015^1^ Viegas et al. 2018^3^ Jaus et al. 2022^3^	See Sueck et al. 2020 for data on kinetics.
Total ochratoxin A (tOTA)	2/6^5^	Orti et al. 1986^1^ Muñoz et al. 2014^2^ Schmidt et al. 2021a^3^	2	Muñoz et al. 2010^3^	Detection after treatment with glucuronidase. See Table 11.
Total ochratoxin α (tOTα)	2	Muñoz et al. 2010^1^ Coronel et al. 2011^3^ Schmidt et al. 2021a^4^	2	Muñoz et al. 2010^1^ Ali et al. 2017b^3^	Intestinal metabolite, prone to misidentification.

^1^First report on the occurrence in humans. ^2^Report on quantitative relationship with exposure. ^3^Example of a recent method where the compound was detected. ^4^Example of a recent method where the compound was not detected. ^5^See text for explanation

Due to the potential relevance of glucuronides of OTA, it is important to note that the correlation reported by (Muñoz et al. 2014) was determined after glucuronidase treatment of urine, whereas the possible sample treatment in the study by Gilbert et al. (2001) is not specified. In contrast, the excretion rate of 2.5% published by Studer-Rohr et al. (2000) was determined without glucuronidase treatment. The critical role of glucuronidase treatment has also been emphasized by Ali et al. (2017b) and should be taken into consideration during method development. Additionally, careful attention should be paid to evaluating the specificity of OTα analysis, particularly when high levels of OTα or high detection rates are observed. In such cases, confirmatory analysis by other experienced laboratories or analysis of the ³⁷Cl isotopomers is strongly recommended.

### Citrinin

Following ingestion, CIT is absorbed and can be detected in human blood as both the parent compound and its main human metabolite, dihydrocitrinone (DH-CIT) (Blaszkewicz et al. 2013; Ali and Degen 2019). While the first methods for detecting CIT in serum and urine were published in the 1980s (Phillips et al. 1980), the first report on CIT in human blood and urine was not made until more than 30 years later by Njumbe Ediage et al. (2012) and Blaszkewicz et al. (2013), who already included DH-CIT. Since then, several multi-metabolite methods have been published, covering these compounds, with some examples of recently applied methods listed in Table 3.

CIT and DH-CIT in urine represent, on average, 40% of the CIT uptake, based on a study involving two individuals. In the same study, a plasma clearance of 7.2 mL/min was determined, resulting in a median biological half-life of 9.4 h (Degen et al. 2018). The CIT/DH-CIT ratio varied significantly in this study, indicating that analysing both compounds improves data quality and accuracy of exposure assessment. Despite the limitation of only two individuals, CIT and DH-CIT appear to be suitable biomarkers for exposure assessment based on urine and blood analysis (Ali and Degen 2019).

### Zearalenone

ZEN is a mycotoxin that undergoes extensive metabolism after ingestion, as demonstrated by numerous in vitro and in vivo studies reviewed by Llorens Castelló et al. (2022). For biomonitoring purposes in humans, the phase 1 metabolism of ZEN to α- and β-zearalenol (α-ZEL/β-ZEL) and the phase 2 metabolism to glucuronides and sulfates are of particular interest (see Table 4). Additionally, the saturated form zearalanone (ZAN) and the corresponding alcohols α- and β-zearalanol (α-ZAL/β-ZAL) and their phase 2 metabolites are covered by several analytical methods and were indeed the first ZEN derivatives monitored in human urine as part of doping analyses as α-ZAL is also used as a growth hormone and applied in cattle as the drug Ralgro^®^ (Ayotte et al. 1996). Due to the importance of phase 2 metabolites, treatment with glucuronidase and arylsulfatase is often applied during the analysis of urine and blood samples to reduce the number of required reference standards and to improve sensitivity.

However, human in vivo data on ZEN metabolism and excretion are limited to two studies. One study involved administration of a very high dose of ZEN, while the other study monitored ZEN exposure in a single male volunteer over four consecutive days (Mirocha et al. 1981; Warth et al. 2013a). In both studies, samples were treated with glucuronidase, but only the first experiment analysed α-ZEL and β-ZEL in addition to ZEN. Based on these data, it was estimated that 7.0–13.2% of ZEN is excreted within 24 h via urine as ZEN and ZEN conjugates, while 10–20% of the dose is excreted as the sum of ZEN, α-ZEL, β-ZEL, and their conjugates (Metzler et al. 2010). However, both studies have limitations, and uncertainties are further increased due to individual differences in ZEN metabolism to the corresponding alcohols, as reported by Ali and Degen (2018). Due to these limitations, several studies have used kinetic data from a feeding experiment with piglets to assess exposure, which showed that 28.4% of ingested ZEN was excreted as total ZEN (tZEN) and 8.3% as tZEN conjugates (Gambacorta et al. 2013). This highlights the need for further research to better understand ZEN metabolism and excretion in humans.

**Table 4:** Zearalenone (ZEN) and metabolites and their application for mycotoxin exposure assessment (Categories (Cat) according to Table 1, n/a: not applicable)

	Urine		Blood/serum/plasma		Comment
	Cat	Reference	Cat	Reference	
Zearalenone (ZEN)	4	Bandera et al. 2011^1^ Cirlini et al. 2017^3^ Li et al. 2018^3^	2	Fan et al. 2019^1^ Vidal et al. 2021^3^ Ning et al. 2024^3^	Urinary biomarker in combination with α-ZEL and β-ZEL. See Table 11.
α-Zearalenol (α-ZEL)	4	Bandera et al. 2011^1^ Abia et al. 2013^3^ Li et al. 2018^3^	1	De Ruyck et al. 2020^3^ Ning et al. 2024^3^ del Delgado-Povedano et al. 2025^4^	Urinary biomarker in combination with β-ZEL and ZEN. See Table 11.
β-Zearalenol (β-ZEL)	4	Bandera et al. 2011^1^ Njumbe Ediage et al. 2012^3^ Li et al. 2018^3^	1	De Ruyck et al. 2020^1^ Tesfamariam et al. 2022^3^ Ning et al. 2024^4^	Urinary biomarker in sum with α-ZEL and ZEN. See Table 11.
Zearalanone (ZAN)	2	Bandera et al. 2011^1^ Li et al. 2018^3^ Fan et al. 2019^3^	1	Fan et al. 2019^1^ Tesfamariam et al. 2022^3^ Ning et al. 2024^4^	
α-Zearalanol (α-ZAL)	1	Bandera et al. 2011^1^ Zhang et al. 2020^4^ Fan et al. 2019^4^	1	Fan et al. 2019^4^ Ning et al. 2024^1^ del Delgado-Povedano et al. 2025^4^	Growth hormone. In some countries applied for cattle (e.g. Ralgro^®^).
β-Zearalanol (β-ZAL)	1	Bandera et al. 2011^1^ Martíns et al. 2019^4^ Fan et al. 2019^4^	1	Tesfamariam et al. 2022^1^ Fan et al. 2019^4^ Ning et al. 2024^4^	
ZEN-14-glucuronide (ZEN-14-GlcA)	2	Warth et al. 2012a^1^ Cirlini et al. 2017^3^ Martíns et al. 2019^3^	0	Fan et al. 2019^4^ De Ruyck et al. 2020^4^	Urinary biomarker in sum with ZEN, and α-/β-ZEL.
ZAN-14-glucuronide (ZAN-14-GlcA)	0	Gerding et al. 2014^4^ Heyndrickx et al. 2015^4^ Fan et al. 2019^4^	0	Fan et al. 2019^4^	
α-ZEL-7-glucuronide (α-ZEL-7-GlcA)	0	Heyndrickx et al. 2015^4^	n/a		
α-ZEL-14-glucuronide (α-ZEL-14-GlcA)	0	Gerding et al. 2014^4^ Heyndrickx et al. 2015^4^	n/a		
β-ZEL-14-glucuronide (β-ZEL-14-GlcA)	1	Gerding et al. 2014^4^ Heyndrickx et al. 2015^1^ Kyei et al. 2022^4^	n/a		
ZEN-14-sulfate	1	Cirlini et al. 2017^4^ Martíns et al. 2019^4^ Kyei et al. 2022^1^	n/a		
Total zearalenone (tZEN)	6	Shephard et al. 2013^1^ Warth et al. 2013a^2^ (Föllmann et al. 2016)^3^	n/a		Urinary biomarker. Data based on a single individual (Mirocha et al. 1981). See Table 11.
Total zearalanone (tZAN)	1	Li et al. 2018^1^ Zhang et al. 2020^4^ McKeon et al. 2024^4^	n/a		
Total α-zearalenol (t-α-ZEL)	4	Shephard et al. 2013^1^ Solfrizzo et al. 2014^3^ McKeon et al. 2024^3^	n/a		Urinary biomarker in combination with tZEN and t-β-ZEL. Data based on a single individual. See Table 11.
Total β-zearalenol (t-β-ZEL)	4	Shephard et al. 2013^1^ Solfrizzo et al. 2014^3^ Li et al. 2018^3^	n/a		Urinary biomarker in combination with tZEN and t-α-ZEL. Data based on a single individual. See Table 11.
Total α-zearalanol (t-α-ZAL)	1	Belhassen et al. 2015^1^ Li et al. 2018^3^ Schmidt et al.2021a^4^	n/a		Only α-ZAL quantified by Belhassen et al. (2015). Application as growth promoter in cattle as source proposed.
Total β-zearalanol (t-β-ZAL)	0	Belhassen et al. 2015^1^ Li et al. 2018^3^ Schmidt et al. 2021a^4^	n/a		

^1^First report on the occurrence in humans. ^2^Report on quantitative relationship with exposure. ^3^Example of a recent method where the compound was detected. ^4^Example of a recent method where the compound was not detected

### Deoxynivalenol and other type B trichothecenes

Following ingestion, DON is rapidly absorbed, and 95% of the toxin is excreted within 12 h, with the highest urinary excretion rate occurring between 30 min and 1 h after administration (Mengelers et al. 2019). However, in this study the bolus was administered to an empty stomach and the kinetic data might not reflect the real situation (Vidal et al. 2018a). Approximately 64.0 ± 22.8% of the toxin dose is excreted renally in its unmodified form or as a glucuronic acid conjugate (Warth et al. 2013a; Vidal et al. 2018a). Specifically, DON-15-GlcA accounts for 58.2% ± 8.7% of excreted DON, while unmodified DON and DON-3-glucuronide (DON-3-GlcA) account for 27.4 ± 11.8% and 14.4 ± 6.7%, respectively (Vidal et al. 2018a). Additional metabolites of DON include deepoxy-deoxynivalenol (DOM-1) as well as DOM-1–3-glucuronide (DOM-1–3-GlcA) and DON-3-sulfate (Turner et al. 2010a; Warth et al. 2016b; Vidal et al. 2018a). Strong correlations between DON exposure as well as cereal consumption and urinary total DON were described in several cohort studies (Turner et al. 2008a, b, 2010b).

Besides DON, the acetylated forms 15-Ac-DON and 3-Ac-DON, as well as the plant metabolite DON-3-glucoside, are commonly present in food, albeit at lower concentrations. Studies have shown that these derivatives are almost quantitatively converted into DON and excreted in a comparable range. However, when DON-3-glucoside was administered to a cohort, approximately 4% of excreted DON was recovered as DON-3-glucoside (Vidal et al. 2018a). DOM-1 is the de-epoxy metabolite of DON formed by gut bacteria following DON ingestion and is regarded as a detoxification product (Pestka and Smolinski 2005). DOM-1 is occasionally observed in human urine (Turner et al. 2010a; Vidal et al. 2016; Deng et al. 2018), but it has not been validated as a biomarker of DON exposure.

Despite the rapid elimination of DON, a few studies have reported the presence of DON, DON-glucuronides, and 3-Ac-DON in blood. However, due to the fast elimination, these bio-measures are not suitable for HBM (Fan et al. 2019; De Ruyck et al. 2020; Tesfamariam et al. 2022; Ning et al. 2024).

As part of the European Biomonitoring Initiative HBM4EU, the suitability of analytical methods for the determination of DON exposure via urine was further elaborated and it was concluded that either glucuronidase treatment to determine the sum of free and conjugated DON (tDON) or the analysis of DON and at least DON-15-GlcA should be applied (Sabbioni et al. 2022).

The presence of nivalenol (NIV) in human urine has been confirmed by a few research groups, including methods applying glucuronidase treatment to determine total NIV (tNIV) as listed in Table 5. Fusarenone X (FUSX), the at position 4 acetylated form of NIV, has not yet been detected in urine samples, but in plasma it has been reported in three studies using two different methods (De Ruyck et al. 2020; Ning et al. 2024; Tesfamariam et al. 2022).

Little is known about the metabolism and bioavailability of FUSX and NIV in humans. Based on data from animal studies, both generally have a low bioavailability and besides the conversion of FUSX to NIV, DOM-1 is the only reported metabolite which is expected to be mostly formed by the intestinal microbiota (Onji et al. 1989).

**Table 5:** Deoxynivalenol (DON), nivalenol (NIV), fusarenone x (FUSX) and metabolites and their application for mycotoxin exposure assessment (Categories (Cat) according to Table 1, n/a: not applicable)

	Urine		Blood/serum/plasma		Comment
	Cat	Reference	Cat	Reference	
Deoxynivalenol (DON)	6	Warth et al. 2011^1,2^ Gerding et al. 2014^3^ Heyndrickx et al. 2015^3^	1	Fan et al. 2019^1,3^ Ning et al. 2024^3^ del Delgado-Povedano et al. 2025^3^	First report of DON as tDON by Meky et al. 2003 Urine: Sum with DON-15-GlcA and DON-3-GlcA recommended biomarker. See Table 11.
DON-3-glucuronide (DON-3-GlcA)	6	Warth et al. 2011^1,2^ Gerding et al. 2014^3^ Heyndrickx et al. 2015^3^	1	De Ruyck et al. 2020^1,3^ Tesfamariam et al. 2022^3^ Ning et al. 2024^4^	Urine: Sum with DON-15-GlcA and DON recommended biomarker. See Table 11.
DON-15-glucuronide (DON-15-GlcA)	6	Warth et al. 2012b^1,2^ Heyndrickx et al. 2015^3^ Vidal et al. 2016^3^	0	De Ruyck et al. 2020^4^ Tesfamariam et al. 2022^4^	Urine: Sum with DON and DON-3-GlcA recommended biomarker. See Table 11.
DON-3-sulfate	1	Warth et al. 2016b^3^	n/a		
Deepoxy-deoxynivalenol (DOM-1)	2	Turner et al. 2010a^1^ Vidal et al. 2016^3^ Deng et al. 2018^3^	1	De Ruyck et al. 2020^1,3^ Tesfamariam et al. 2022^3^ Ning et al. 2024^4^	Only small amounts formed.
DOM-1-glucuronides (DOM-1-GlcA)	1	Heyndrickx et al. 2015^1^	n/a		Only small amounts formed.
3-Acetyl-deoxynivalenol (3-Ac-DON)	1	Heyndrickx et al. 2015^4^ Vidal et al. 2016^1^ Fan et al. 2019^4^	1	Tesfamariam et al. 2022^3^ Ning et al. 2024^3^ del Delgado-Povedano et al. 2025^4^	Parent compound; only small quantities are not deacetylated
15-Acetyl-deoxynivalenol (15-Ac-DON)	0	Heyndrickx et al. 2015^4^ Deng et al. 2018^4^ De Ruyck et al. 2020^4^	0	Fan et al. 2019^4^ Ning et al. 2024^4^ del Delgado-Povedano et al. 2025^4^	Parent compound; only small quantities are not deacetylated
15-Ac-DON-3-glucuronide (15-Ac-DON-3-GlcA)	0	Heyndrickx et al. 2015^4^	n/a		
DON-3-glucoside (DON-3-G)	4	Vidal et al. 2018a^1^ Martíns et al. 2019^3^ De Ruyck et al. 2020^4^	0	De Ruyck et al. 2020^4^ Vidal et al. 2021^4^ Tesfamariam et al. 2022^4^	Parent compound; only small quantities are not deglucosidated
total deoxynivalenol (tDON)	6	Meky et al. 2003^1^ Turner et al. 2010a^2^ Schmidt et al. 2021a^3^	n/a		Urine: Recommended biomarker besides direct analysis of DON and glucuronides. See Table 11.
total deepoxy-deoxynivalenol (tDOM-1)	2	Turner et al. 2010a^1,2^ Föllmann et al. 2016^3^ (Xia et al. 2022)^3^	n/a		Only small amounts formed.
Nivalenol (NIV)	2	Warth et al. 2012b^1,3^ Shephard et al. 2013^3^ De Ruyck et al. 2020^3^	1	De Ruyck et al. 2020^3^ Tesfamariam et al. 2022^3^ Del Mar Delgado-Povedano et al.^2025^^4^	
total nivalenol (tNIV)	2	Wallin et al. 2015^1,3^ Šarkanj et al. 2018^3^ Abia et al. 2024^3^	n/a	n/a	
Fusarenon X (FUSX)	0	Heyndrickx et al. 2015^4^ Fan et al. 2019^4^	1	De Ruyck et al. 2020^3^ Ning et al. 2024^3^ Tesfamariam et al. 2022^3^ Del Mar Delgado-Povedano et al.^2025^^4^	

^1^First report on the occurrence in humans. ^2^Report on quantitative relationship with exposure. ^3^Example of a recent method where the compound was detected. ^4^Example of a recent method where the compound was not detected

### Fumonisins

Fumonisins B_1_–B_3_ (FB1-FB3) are poorly absorbed, and the limited bioavailable fraction is mostly biliary-excreted without modification, resulting in a urinary excretion rate of FB1 below 1% in humans and between 0.4 and 2.6% in experimental animals (Turner and Snyder 2021; Szabó-Fodor et al. 2022). Even lower excretion rates have been observed for FB2 and FB3 (Torres et al. 2014). Currently, two independent studies form the basis for FB1 exposure assessment via urinary excretion rates (van der Westhuizen et al. 2013; Riley et al. 2012). van der Westhuizen et al. (2011) analysed first-void urine samples from 22 female participants in South Africa and estimated an excretion rate of 0.075% (range: 0.054%–0.104%) based on FB1 uptake via food on the previous day. These data were collected as part of an intervention study with two urine samplings at relatively high exposure (baseline) and two at lower exposure (intervention) to FB1. Riley et al. (2012) investigated the kinetics of urinary FB1: in a study involving a single individual, the authors analysed all urine excreted and determined an excretion rate of 0.78% for FB1. In a second study with eight individuals, analysis of evening urine samples (where the highest FB1 concentrations in urine were found) yielded an excretion rate of 0.50% ± 0.24%. The biological half-life of FB1 was estimated to be slightly under 48 h for short-term exposure (3 days) and between 48 and 72 h for longer exposure periods (6 days) (Riley et al. 2012). The differences between the determined excretion rates for FB1 can have several causes, with the timing of urine sampling being particularly relevant. Additional factors, such as cohort differences, dietary habits, and timing of food intake, may also contribute, as the studies are based on spot urine samples. For exposure assessment, a mean value between the two rates is often used to estimate exposure. Besides the uncertainty related to urinary excretion rates, a significant (mostly *p* < 0.01), but only low-moderate correlation (r² = 0.26) between FB1 intake and observed urinary concentrations is another limitation (Torres et al. 2014; Turner and Snyder 2021). Where this measure is to be used in epidemiological studies, the poor exposure estimate (based on the r-squared value above) means that careful consideration is needed in sample size calculations (Turner and Snyder 2021). Urinary excretion of FB2 and FB3 is significantly lower than that of FB1; thus, exposure assessment based on urine concentrations is not yet possible for these compounds (Riley et al. 2012; Torres et al. 2014).

Fumonisins are ceramide synthase inhibitors, blocking the *N*-acylation of sphinganine (Sa) to dihydroceramide. This results in elevated levels of Sa in tissues and, to a much lesser extent, sphingosine (So), which is not a precursor for *de novo* ceramide synthesis (Merrill et al. 2001; Riley et al. 2001). Increased cellular levels of free Sa are converted to sphinganine-1-phosphate (Sa 1-P), which accumulates in tissues and body fluids. Consequently, monitoring alterations in sphingolipid metabolism has been proposed as a possible biomarker of effect for FB1 exposure. Initial studies focused on the Sa/So ratio but could demonstrate changes only at very high exposure scenarios, as reviewed by van der Westhuizen (2013). In contrast, a correlation between Sa 1-P concentration (r² = 0.27, *p* < 0.001), and even more strongly for the Sa 1-P/So 1-P ratio (r² = 0.49, *p* < 0.001) in dried blood spot (DBS)-samples, with urinary FB1 was demonstrated (Riley et al. 2015a, (Riley, et al., b)). Based on the correlation data, the authors concluded that a urinary FB1 breakpoint concentration between 0.5 and 1.0 ng/mL is required to observe changes in the Sa 1-P/So 1-P ratio. Thus, analysis of DBS for the Sa 1-P/So 1-P ratio might be a possibility for blood-sample based assessment of FB1 exposure. Fumonisins, their metabolites, and sphingolipid biomarkers are summarized in Table 6.

**Table 6:** Fumonisins, potential biomarkers of effect and their application for mycotoxin exposure assessment (Categories (Cat) according to Table 1, n/a: not applicable)

	Urine		Blood/serum/plasma		Comment
	Cat	Reference	Cat	Reference	
Fumonisin B_1_ (FB1)	6	Gong et al. 2008^1,2^ van der Westhuizen et al. 2011^2^ Riley et al. 2012^2^ Abia et al. 2024^3^	2	Cao et al. 2018^1,3^ De Ruyck et al. 2020^3^ Ning et al. 2024^3^	Urinary biomarker. See Table 11.
Fumonisin B_2_ (FB2)	2	Warth et al. 2012a^1^ Torres et al. 2014^3^ De Ruyck et al. 2020^3^	2	Cao et al. 2018^1,3^ De Ruyck et al. 2020^3^ Ning et al. 2024^3^	
Fumonisin B_3_ (FB3)	1	Torres et al. 2014^1^ Martíns et al. 2019^4^ De Ruyck et al. 2020^1,3^	2	De Ruyck et al. 2020^1,3^ Tesfamariam et al. 2022^3^ Ning et al. 2024^3^	
Hydrolyzed fumonisin B_1_ (HFB1)	0	Heyndrickx et al. 2015^4^ Martíns et al. 2019^4^ De Ruyck et al. 2020^4^	0	Heyndrickx et al. 2015^4^ De Ruyck et al. 2020^4^ Tesfamariam et al. 2022^1,3^	
Sphinganine (Sa), sphingosine (So), Sa/So-ratio	n/a	Riley et al. 1994^1^ Castegnaro et al. 1996 Silva et al. 2009	1	Riley et al. 1994^1^	Analysis in exfoliated cells in urine. Not suitable as biomarker in humans.
Sphinganine-1-phosphate (Sa 1-P), sphingosine-1-phosphate (So 1-P), Sa 1-P/So 1-P-ratio	n/a		4	Riley et al. 2015a^1,2^ Riley et al. 2015b^3^	Red blood cells required, analysis only for dried blood spots published.

^1^First report on the occurrence in humans. ^2^Report on quantitative relationship with exposure. ^3^Example of a recent method where the compound was detected. ^4^Example of a recent method where the compound was not detected

### *Alternaria* toxins

To date, the mycotoxins alternariol (AOH), alternariol monomethyl ether (AME), altenuene (ALT), altertoxins I-III (ATX), tentoxin (TEN), and tenuazonic acid/*allo*-tenuazonic acid (TeA/*allo*-TeA) have been incorporated into HBM methods (Table 7). AOH and AME are frequently included in multi-metabolite methods, and occasional detections of one or both compounds at low levels have been published for serum (Tesfamariam et al. 2022; Ning et al. 2024) and more often in urine (Martins et al. 2019; De Ruyck et al. 2020; Fan et al. 2021; Qiao et al. 2022). ATX and ALT have not yet been detected in blood, despite being included in multiple methods (De Ruyck et al. 2020; Ning et al. 2024). In urine besides a few samples (< 0.5%) positive for ALT in one study, no detects are reported for these mycotoxins (Qiao et al. 2022). Presence of TEN in serum and urine has so far only been reported by few laboratories (Ning et al. 2024; Fan et al. 2021; Qiao et al. 2022).

In contrast to the other *Alternaria* toxins, since the first analytical method was published by Asam et al. (2013), TeA is frequently detected in urine, when included in analytical methods (Hövelmann et al. 2016; De Ruyck et al. 2020; Fan et al. 2021; Schmidt et al. 2021a, b; Qiao et al. 2022). Low levels of TeA have also been reported in serum by two different laboratories (Tesfamariam et al. 2022; Delgado-Povedano et al. 2025).

TeA may undergo racemization during food processing, such as heat treatment of tomato puree, resulting in the formation of *allo*-TeA. This compound has nearly identical chromatographic and mass spectrometric properties, making it challenging to separate from TeA by ultra-high-performance liquid chromatography (UHPLC). Hövelmann et al. (2016) found that *allo*-TeA accounted for 3.4–25.0% of total urinary TeA in a German cohort. However, the absorption, distribution, metabolism, and excretion of *allo*-TeA have not been studied while first in vitro data indicate that *allo-*TeA is less cytotoxic (Hövelmann et al. 2016). It is also unclear if *allo*-TeA can be formed from TeA under physiological conditions in the human body.

A review on the hazard characterization of *Alternaria* toxins has recently been published, summarizing available data on the distribution, metabolism, and excretion of these compounds (Louro et al. 2024). So far only in vivo data on TeA excretion in humans are available from two independent studies. A study monitoring urinary TeA excretion after consumption of natural contaminated food resulted in an excretion rate of 89 ± 4% within 24 h (Asam et al. 2013). In contrast, Visintin et al. (2025) determined an excretion rate of only 32 ± 17% with the last detectable urinary concentrations 13 h past ingestion and a biological half-life of only 1.9 h, leading to a detection window in blood of about 6 h from exposure. The authors were also able to determine an average blood clearance rate of 1.56 L/(hr⋅kg) with a 90% confidence interval for the population ranging from 1.15 to 2.04 L/(hr⋅kg). Additionally, enzymatic treatment performed on both blood and urinary samples yielded higher concentrations in comparison with samples prepared without hydrolysis, suggesting that TeA undergoes phase II metabolism.

In contrast to Asam et al. (2013), the study by Visintin et al. (2025) was performed under highly controlled conditions with a single dose, resulting in a comprehensive data set, but due to the absence of food matrix, the kinetic data might not reflect the real situation. The confirmation of a linear relationship between TeA exposure and excretion still needs to be assessed.

**Table 7:** Analytes of the *Alternaria*-group and their application for mycotoxin exposure assessment (Categories (Cat) according to Table 1, n/a: not applicable)

	Urine		Blood/serum/plasma		Comment
	Cat	Reference	Cat	Reference	
Alternariol (AOH)	3	Šarkanj et al. 2018^1^ Martíns et al. 2019^3^ De Ruyck et al. 2020^3^	1	Tesfamariam et al. 2022^1^ Ning et al. 2024^3^	
Alternariol monomethylether (AME)	1	De Ruyck et al. 2020^1^ Fan et al. 2021^3^ Qiao et al. 2022^3^	2	Tesfamariam et al. 2022^1^ Ning et al. 2024^3^ De Delgado-Povedano et al. 2025^4^	
Altenuene (ALT)	1	De Ruyck et al. 2020^4^ Fan et al. 2021^4^ Qiao et al. 2022^1^	0	Osteresch et al. 2017^4^ De Ruyck et al. 2020^4^ Ning et al. 2024^4^	
Altertoxins (ATX)	0	De Ruyck et al. 2020^4^	0	Ning et al. 2024^4^	
Tentoxin (TEN)	n/a	Fan et al. 2021^1^ Qiao et al. 2022^3^	1	Ning et al. 2024^1^	
Tenuazonic acid (TeA)	6	Asam et al. 2013^1,2^ Hövelmann et al. 2016^3^ Visintin et al. 2025^2,3^	2	Tesfamariam et al. 2022^1^ Delgado-Povedano et al. 2025^3^ Visintin et al. 2025^3^	See Table 11.
*allo*-tenuazonic acid (*allo*-TeA)	2	Hövelmann et al. 2016^1^ Schmidt et al. 2021a^3^	n/a		*allo-*tenuazonic acid used to coelute wit TeA under most RP-HPLC conditions.

^1^First report on the occurrence in humans. ^2^Report on quantitative relationship with exposure. ^3^Example of a recent method where the compound was detected. ^4^Example of a recent method where the compound was not detected

### T-2 toxin and other type A trichothecenes

The type A trichothecenes currently covered by HBM methods are summarized in Table 8. These toxins are produced by various *Fusarium* species, with T-2 toxin (T-2) and HT-2 toxin (HT-2) being the compounds most commonly detected in food. The first reports of T-2 and HT-2 occurrence in human biological samples date back to 1983, when Mirocha et al. identified these toxins in urine and blood samples from victims of war in Laos and Southeast Asia (Mirocha et al. 1983). Although the hypothesis that T-2 and HT-2 were used as chemical warfare agents has since then been largely refuted, the general presence of T-2 in human samples has been confirmed (Black 2010; Marshall 1986).

Based on animal experiments and in vitro studies using human liver microsomes, T-2 undergoes various forms of biotransformation (Wu et al. 2020). These include deacetylation to HT-2 and T-2 triol (Triol), cleavage of the isovaleric acid side chain to form neosolaniol (NEO) and T-2 tetraol (Tetraol), as well as hydroxylation at the isovaleric acid group to yield 3′-hydroxy-T-2 (3′-OH-T-2), 3′-hydroxy-HT-2 (3′-OH-HT-2), 4′-hydroxy-T-2 (4′-OH-T-2), and 4′-hydroxy-HT-2 (4′-OH-HT-2)(Corley et al. 1986; Weidner et al. 2012; Yang et al. 2013, 2017). In these experiments, also glucuronides of T-2 and its metabolites were detected.

Analysis of human urine samples by HPLC-MS/MS for type A trichothecenes started with Gerding et al. (2014) who was able to detect T-2 in very few samples from Germany. Subsequently, with improved limits of detection (LODs) also HT-2, diacetoxyscirpenol (DAS), NEO, and verrucarol (VER) were found in low frequencies and only by one or two laboratories (De Ruyck et al. 2020; Narváez et al. 2021). For human plasma, detections of T-2, HT-2, DAS, and 15-acetoxyscirpenol are occasionally reported (De Ruyck et al. 2020; Tesfamariam et al. 2022; Ning et al. 2024).

While some glucuronides were tentatively identified in human urine by UHPLC-HRMS in the absence of reference standards, a convincing demonstration of the presence of glucuronides of HT-2 and Triol in human urine was achieved by McKeon et al. (2025), who compared samples with (tT-2, tHT-2, tTriol) and without (T-2, HT-2, Triol) treatment with β-glucuronidase from *E. coli.* In their study no T-2, tT-2, HT-2 or Triol were detected but ≥ 99% of urine samples were positive for tHT-2 and two samples from one participant positive for tTriol.

No data on T-2/HT-2 kinetics or biotransformation in humans are published where a defined dose of the mycotoxins was administered to participants. In animals, several feeding studies have been conducted, as reviewed by Van den Brand and Mengelers (2021). One early study was conducted in swine, in which two animals received radiolabelled T-2 intravascularly, resulting in a plasma half-life estimate of 1.5 h (Corley et al. 1986).

McKeon et al. (2025) were able to derive excretion and clearance data from a cohort study involving 40 participants who provided weighed 24-h dietary records and 24-hour urine samples, collected at three time windows: 06:00–12:00, 12:00–18:00, and 18:00–08:30 h. With ≥ 97% of samples containing quantifiable amounts of tHT-2, they were able to align food consumption data with tHT-2 excretion as T-2, Triol and tT-2 were not detected. Based on data from 32 participants, a mean residence time of 4.9 h and a median half-life of 4.0 h were determined. The fraction of T-2/HT-2 excreted as tHT-2 was calculated at 18.4%; however, it must be noted that no food samples were analysed, and exposure was estimated from published food contamination data, which introduces substantial uncertainty.

**Table 8:** Type A trichothecenes, their metabolites and application for mycotoxin exposure assessment (Categories (Cat) according to Table 1, n/a: not applicable)

	Urine		Blood/serum/plasma		Comment
	Cat	Reference	Cat	Reference	
T-2 toxin (T-2)	2	Mirocha et al. 1983^1^ Gerding et al. 2014^3^ De Ruyck et al. 2020^3^	2	Mirocha et al. 1983^1^ De Ruyck et al. 2020^3^ Tesfamariam et al. 2022^3^	
HT-2 toxin (HT-2)	1	Mirocha et al. 1983^1^ De Ruyck et al. 2020^3^	2	Mirocha et al. 1983^1^ De Ruyck et al. 2020^3^ Tesfamariam et al. 2022^3^ Ning et al. 2024^4^	
T-2 triol (Triol)	1	Martíns et al. 2019^4^ De Ruyck et al. 2020^1^	0	De Ruyck et al. 2020^4^ Tesfamariam et al. 2022^4^ Ning et al. 2024^4^	
T-2 tetraol (Tetraol)	1	Martíns et al. 2019^4^ De Ruyck et al. 2020^1^	0	De Ruyck et al. 2020^4^ Tesfamariam et al. 2022^4^	
Neosolaniol (NEO)	1	Martíns et al. 2019^4^ De Ruyck et al. 2020^1^ Narváez et al. 2021^3^	2	De Ruyck et al. 2020^1^ Tesfamariam et al. 2022^3^ Ning et al. 2024^3^	
T-2–3-glucuronide (T-2–3-GlcA)	1	Narváez et al. 2021^1*^ Kyei et al. 2022^4^	n/a	n/a	
HT-2–3-glucuronide (HT-2–3-GlcA)	1	Narváez et al. 2021^1*^	n/a	n/a	
HT-2–4-glucuronide (HT-2–4-GlcA)	0	Gerding et al. 2014^4^ Narváez et al. 2021^4^ Kyei et al. 2022^4^	0	Osteresch et al. 2017^4^	
Total T-2 toxin (tT-2)	0	Franco et al. 2019^4^ Schmidt et al. 2021a^4^ Gallardo-Ramos et al. 2024^4^ Mc Keon et al. 2025^4^	n/a	n/a	
Total HT-2 toxin (tHT-2)	1(4)	Schmidt et al. 2021a^4^ Gallardo-Ramos et al. 2024^4^ Mc Keon et al. 2025^1,2^	n/a	n/a	Most promising biomarker candidate for T-2/HT-2 exposure assessment. See Table 11.
Total T-2 triol (tTriol)	1	Mc Keon et al. 2025^1^	n/a	n/a	
Diacetoxyscirpenol (DAS)	1	Heyndrickx et al. 2015^4^ Martíns et al. 2019^4^ De Ruyck et al. 2020^1^	2	De Ruyck et al. 2020^1^ Tesfamariam et al. 2022^3^ Ning et al. 2024^3^	
15-Acetoxyscirpenol	n/a	n/a	1	Ning et al. 2024^1^	
Verrucarol (VER)	1	De Ruyck et al. 2020^1^	0	De Ruyck et al. 2020^4^	

^1^First report on the occurrence in humans. ^2^Report on quantitative relationship with exposure. ^3^Example of a recent method where the compound was detected. ^4^Example of a recent method where the compound was not detected

### Other mycotoxins

#### Beauvericin and enniatins

Beauvericin (BEA) and enniatins, namely enniatin B (EnB), enniatin B_1_ (EnB1), enniatin A (EnA) and enniatin A_1_ (EnA1) are cyclohexadepsipeptide mycotoxins produced by different *Fusarium* species (Fraeyman et al. 2017). These compounds are cytotoxic, and the effects seem to be related to their ionophoric properties (EFSA 2014a). Their bioavailability in humans is expected to vary as a pilot study with a pig receiving an oral bolus reported clear differences in the maximum plasma concentration of the different enniatins (EnB > EnB1 > EnA1 > EnA) and very low BEA levels (Devreese et al. 2013). In a follow-up kinetic study with pigs for EnB1 a high bioavailability of 91%, and a biological half-life of 1.57 ± 0.746 h and a plasma clearance of 2.00 ± 0.637 L/h/kg were determined (Devreese et al. 2014). As no urine samples were taken, the urinary excretion rate was not calculated.

BEA and enniatins are reported to be metabolized by carbonylation, carboxylation, hydroxylation, and *N*-demethylation based on experiments in vitro and in vivo in pigs. However, besides MS-based compound characterization with additional derivatization experiments, no structure elucidation of metabolites has been performed (Ivanova et al. 2011, 2017). Qualitative analysis of human urine samples for these metabolites of EnB and EnB1 in human urine samples confirmed the metabolism (Rodríguez-Carrasco et al. 2018, 2020).

However, quantification was so far only possible for the parent compounds with EnB, showing the highest occurrence rates, followed by EnB1 (Gerding et al. 2015; Osteresch et al. 2017). Detections for EnA and EnA1 are comparably rare (Gallardo-Ramos et al. 2024) (Table 9).

**Table 9:** Beauvericin and enniatins and their application for mycotoxin exposure assessment (Categories (Cat) according to Table 1, n/a: not applicable)

	Urine		Blood/serum/plasma	
	Cat.	Ref	Cat.	Ref
Beauvericin (BEA)	1	Serrano et al. 2015^4^ De Ruyck et al. 2020^4^ Gallardo-Ramos et al. 2024^1,3^	1	Serrano et al. 2015^4^ Osteresch et al. 2017^4^ Ning et al. 2024^1,3^
Enniatin A (EnA)	1	Serrano et al. 2015^4^ Escrivá et al. 2017^1,3^ Gallardo-Ramos et al. 2024^3^	0	Serrano et al. 2015^4^ Osteresch et al. 2017^4^ Ning et al. 2024^4^
Enniatin A_1_ (EnA1)	1	Serrano et al. 2015^4^ Gallardo-Ramos et al. 2024^1,3^	1	Serrano et al. 2015^4^ Osteresch et al. 2017^4^ Ning et al. 2024^1,3^
Enniatin B (EnB)	2	Gerding et al. 2014^1,3^ De Ruyck et al. 2020^3^ Escrivá et al. 2017^3^	4	Osteresch et al. 2017^1,3^ Tesfamariam et al. 2022^3^ Ning et al. 2024^3^
Enniatin B_1_ (EnB1)	2	Serrano et al. 2015^1,3^ Escrivá et al. 2017^3^ Gallardo-Ramos et al. 2024^3^	1	Serrano et al. 2015^1^ Osteresch et al. 2017^4^ Ning et al. 2024^3^

^1^First report on the occurrence in humans. ^3^Example of a recent method where the compound was detected. ^4^Example of a recent method where the compound was not detected

#### Ergot alkaloids

The toxicodynamics and toxicokinetics of different ergot alkaloids have been studied in several animal species (summarized in (EFSA 2012a). For humans, toxicokinetic data are available for ergometrine and ergotamine as they were used as pharmaceuticals in the past. In a human study (*n* = 6), 62 ± 3% of an ingested dose of tritium labelled ergotamine was absorbed and the mean urinary elimination half-life of ergotamine was 31 ± 5 h (Aellig and Nüesch 1977). Sanders et al. (1986) investigated the pharmacokinetics of ergotamine (2 mg) in healthy volunteers (*n* = 24) after oral and rectal dosing. After oral dosing the maximum concentration in plasma was 21.4 ± 2.5 pg/mL reached at 69 ± 39 min. As many time points were near the LOD after oral dosing, the pharmacokinetic data were calculated based on rectal dosing leading to an absorption half-life of 10.5 ± 4.5 min and an elimination half-life of 3.35 ± 0.53 h (Sanders et al. 1986). In another study, 6 male human volunteers were exposed orally and intravenously to 0.200 mg and 0.075 mg ergometrine maleate, respectively (corresponding to 0.147 mg and 0.055 mg ergometrine). Large individual variations were observed and the peak concentrations in plasma following oral exposure ranged from 0.61 to 1.39 µg/l (mean: 1.07 ± 0.29 µg/l). The pharmacokinetic profile can be described by a one-compartment model with an absorption half-life of 0.19 ± 0.22 h and the elimination half-life of 1.9 ± 0.16 h (De Groot et al. 1994).

Oxidative metabolism of ergot alkaloids was shown for ergotamine and ergocristine by in vitro studies in two human cell lines (Mulac et al. 2011). Besides the (di)hydroxylation at positions 8’, 9’ or 10’ of the proline ring, hydroxylation at the ergoline ring leading to 12-hydroxy-ergometrine was detected in rats after i.v. injection of ergometrine (3 mg/kg bw) (Slaytor and Wright 1962).

Analysis of ergot alkaloids in physiological samples is so far limited to the parent compounds and a study by De Ruyck et al. (2020) who report the detection of almost all major ergot alkaloids (ergocornine, ergocorninine, ergocristine, ergocristinine, ergocryptine, ergocryptinine, ergometrine, ergometrinine, ergosine, ergosinine, ergotamine, ergotaminine) in urine as well as in serum samples from the EFCOVAL study, which covered individuals from Belgium, the Czech Republic, France, the Netherlands, and Norway. Detection of ergocorninine, ergocristinine, dihydroergocristine, ergometrine, ergosinine, agroclavine and dihydrolysergol in serum was also reported from the analysis of samples from a cohort in China (Ning et al. 2024).

#### Mycophenolic acid

Mycophenolic acid (MPA) is a naturally occurring compound produced by several species of *Penicillium* fungi but also a medication applied as immunosuppressant, sold under names such as Myfortic^®^. Besides therapeutic drug monitoring, MPA was so far only analysed and found by Ning et al. (2024) in serum. Due to its application as a drug to prevent rejection after organ transplantation but also to treat autoimmune diseases, care must be taken to differentiate between intended exposure via drugs and non-intended exposure.

#### Patulin

Patulin (PAT) is also included in a few recent HBM methods despite its high reactivity with thiol-containing biomolecules (Fliege and Metzler 1999, 2000). The fast reactivity of PAT was confirmed in vivo and in vitro where no PAT could be detected in human serum after ingestion of PAT containing apple juice and a reduction of PAT levels by 94% within two minutes of incubation of PAT in a human blood sample (Rychlik 2003). All analyses were made by applying stable isotope-labelled PAT as internal standard. Nevertheless Ouhibi et al. (2020) and later De Ruyck et al. (2020) and Ning et al. (2024) reported the presence of patulin (PAT) in plasma, which may require additional verification and could be linked to the low molecular mass of PAT and relatively unspecific fragmentation reactions of the MRM experiments. More promising for future biomonitoring could be the analysis of PAT-GSH reaction products, which have been shown to be formed in vitro in human blood (Rychlik 2003).

#### Roquefortine C

First analysis for roquefortine C, another *Penicillium* toxin, as part of an HBM study was made by Martins et al. (2019) in urine but first detects are reported from De Ruyck et al. (2020) in urine and serum. Since then, no further serum/blood samples were found positive for this compound (Vidal et al. 2021; Ning et al. 2024; Delgado-Povedano et al. 2025).

### Mycotoxins in breast milk

The analysis of mycotoxins and their metabolites in breast milk can provide information on the exposure of both, the mother and the infant, to these compounds (Jonsyn et al. 1995; Micco et al. 1995). The decisive factor is the mother’s burden with mycotoxins in blood and the corresponding transfer into milk (Warth et al. 2016a).

Lipophilic mycotoxins and those with a relatively long plasma half-life are of particular relevance. Accordingly, the most frequently detected mycotoxins in breast milk are AFM1 and OTA, with the first reports on occurrence dating back to 1984 and 1988, respectively (Coulter et al. 1984; Gareis et al. 1988). In areas with high maternal exposure, detection rates of 80–100% were often observed, depending on the analytical method used (Warth et al. 2016a; Sengling Cebin Coppa et al. 2019; Hernández et al. 2021).

First reports on the occurrence of ZEN in breast milk were published in 2014, and subsequently in multiple sample sets and countries (Duarte et al. 2022; Rubert et al. 2014). Interestingly, higher occurrence rates were mostly reported in studies applying ELISA compared to HPLC analyses. Reasons for this may involve, in addition to the partially better sensitivity of ELISA, cross-reactivity of the applied antibodies with ZEN metabolites as well as other breast milk constituents. Moreover, the mother’s exposure is decisive for the concentrations found in breast milk, which often correlates with lower living standards and the application of, compared to HPLC, less expensive ELISA kits for detection (Duarte et al. 2022).

The presence of FB1 in breast milk was first reported for highly exposed mothers in 2014 (Magoha et al. 2014). More recently FB1, FB2 and FB3 were observed in samples from a small mixed cohort and a cohort in rural Ethiopia (Martins et al. 2022; Mesfin et al. 2023). FB1 was also reported in 3% of samples from Brazil (Coppa et al. 2021). So far, no further reports on the occurrence of FBs in breast milk have been published.

Due to improved sensitivity and analyte extension of analytical methods, the spectrum of mycotoxins detected in breast milk has increased, with detected mycotoxins and metabolites listed in Table 10. Of particular note are BEA, enniatins, AOH, and AME. In addition, individual detection of AFB1, OTα, HT-2, CIT, DH-CIT, STC, and OTB have also been reported (Turconi et al. 2004; Rubert et al. 2014; Braun et al. 2018; Braun et al. 2020; Braun et al. 2022; Jamnik et al. 2022; Mesfin et al. 2023; Ayeni et al. 2024). In all cases, the number of positive samples are relatively low, and the detected concentrations are usually within the lowest calibration range (Rubert et al. 2014; Braun et al. 2018, 2020, 2022; Jamnik et al. 2022; Mesfin et al. 2023). By analysing breast milk samples in conjunction with plasma samples from the mother, transfer rates of mycotoxins into milk can be determined as milk-to-plasma ratios. However, it must be taken into account that these ratios may change over time, due to changes in the composition of breast milk (Warth et al. 2016a). A significant correlation (*r* = 0.33) between AFB1 intake and AFM1 excretion was shown in a study with 50 nursing mothers in Nigeria (Adejumo et al. 2013). In another study the transfer of ingested AFB1 into breast milk as AFM1 was estimated to be between 0.09 and 0.43% based on the data from a small cohort study in The Gambia (Zarba et al. 1992). Hassan et al. (2006) compared mean aflatoxin contamination levels in serum of breast-feeding mothers, their children and AFM1 in milk. Based on these data, a mean milk-to-plasma ratio of 0.21 was derived (Degen et al. 2013; Warth et al. 2016a). Recently, a good correlation (*r* = 0.72) of AFM1 in breast milk with AFM1 in urine was detected with the average concentration in urine being 25 times higher than in the mean of the breast milk samples positive for AFM1 (50%) (Tuba et al. 2025).

For OTA the transfer from plasma to milk was investigated by Muñoz et al. (2014), reporting that the milk-to-plasma ratio starts with 0.40 ± 0.26 in the first days (colostrum) and then shifts to 0.15 ± 0.26 from day 15 onwards (Muñoz et al. 2014). For the other mycotoxins detected in breast milk, no milk-to-plasma ratios or transfer rates have been published.

**Table 10:** Mycotoxins detected in human breast milk samples

Mycotoxin	Reference
AFB1	Turconi et al. 2004^1^; Martins et al. 2022³; Ayeni et al. 2024³
AFM1	Coulter et al. 1984^1^; Zarba et al. 1992^2^; Braun et al. 2020³
AFM2	Coulter et al. 1984^1^; Braun et al. 2020^4^
AFG1	Zarba et al. 1992^1^; Braun et al. 2020^4^
AOH	Jamnik et al. 2022^1,3^
AME	Braun et al. 2020^1,3^; Ayeni et al. 2024³
BEA	Braun et al. 2018^1^; Ayeni et al. 2024³
CIT	Ayeni et al. 2024^1^
DH-CIT	Braun et al. 2022^1,3^; Ayeni et al. 2024³
ENA	Rubert et al. 2014^1,3^; Braun et al. 2020^3^; Ayeni et al. 2024³
ENA1	Rubert et al. 2014^1,3^; Braun et al. 2020^3^
ENB	Rubert et al. 2014^1,3^; Braun et al. 2018^3^; Ayeni et al. 2024³
ENB1	Rubert et al. 2014^1^; Braun et al. 2020^3^; Ayeni et al. 2024³
FB1	Magoha et al. 2014^1^; Coppa et al. 2021^3^; Mesfin et al. 2023^3^
FB2	Martins et al. 2022^1,3^
FB3	Martins et al. 2022^1,3^
HT-2	Rubert et al. 2014^1,3^; Braun et al. 2020^4^
NEO	Rubert et al. 2014^1,3^
NIV	Rubert et al. 2014^1^; Braun et al. 2020^4^; Mesfin et al. 2023^3^
OTA	Gareis et al. 1988^1^; Braun et al. 2020^3^
OTB	Braun et al. 2020^1,3^; Ayeni et al. 2024³
OTα	Ayeni et al. 2024^1,3^
STC	Braun et al. 2022^1,3^; Ayeni et al. 2024³
ZEN	Rubert et al. 2014^1,3^; Massart et al. 2016^3^; Braun et al. 2020^3^
α-ZEL	Rubert et al. 2014^1,3^; Martins et al. 2022
β-ZEL	Rubert et al. 2014^1,3^

^1^First report on the occurrence in humans. ^2^Report on quantitative relationship with exposure. ^3^Example of a recent method where the compound was detected. ^4^Example of a recent method where the compound was not detected

## Study design and dietary intake records

The choice of study design will reflect the specific objectives of the planned investigation. This may vary from being purely biomonitoring activities to epidemiological or intervention studies. For the former the study design may be focused on obtaining sufficient samples in order to be representative of the mycotoxin of interest. For example, in France a small study may be sufficient to capture typical DON exposure (relatively common) compared to AFB1 exposure (relatively rare). However, in epidemiological studies researchers need to consider the frequency and range of detection of the biomarker, the rarity of the outcome, and the accuracy of the exposure estimate of a given biomarker. For example, an estimate of intake based on urinary FB1 is significantly less accurate than for DON based on the variance of the transfer (see Sect. Recent trends and challenges in interpretation of human biomonitoring data). Thus, studies on FB1 should seek greater numbers.

The number of human cohorts specifically and solely designed for mycotoxin-related research questions is scarce. Many of these focus primarily on aflatoxins (Rasheed et al. 2021; Xu et al. 2021), while others investigate a broader range of mycotoxins (Heyndrickx et al. 2015; Pero-Gascon et al. 2022). Human cohorts are often set up before specific research questions or purposes are fully defined. Some studies are purely biomonitoring, others are conducting epidemiological research, while others are assessing mitigation efforts. This approach can be strategic, allowing the cohort’s principal investigator to be flexible for emerging research questions, multidisciplinary opportunities, hypothesis generation, multiple uses, and technological advances (Pero-Gascon et al. 2022; Kyei et al. 2022). On the other hand, many cohorts are purpose-built around specific mycotoxin exposures and outcomes, allowing the collection of targeted data and inclusion of specified questionnaires (Watson et al. 2018; Namorado et al. 2024). Yet, standardized questionnaires are often lacking, making comparisons between cohorts challenging. As mycotoxins are a niche field, minimal information requirements should be established and account for selection bias of the participants.

### Observational studies: Cross-sectional vs. Longitudinal

Cross-sectional studies are primarily used for descriptive analysis and generating hypotheses. The goal is to examine mycotoxin prevalence within a population at a certain point in time and to compare outcome differences between exposed and unexposed subjects, or, to examine correlation between exposure concentration and the extent of an outcome measure. Advantages of a cross-sectional study are the cost- and time-effectiveness as all variables are only collected once. Although they are relatively easy and quick to conduct, they allow the study of multiple outcomes and exposures at the same time. Cross-sectional study designs are unable to measure incidence and are susceptible to selection and recall bias (Levin 2005a, 2006). Cross-sectional studies provide valuable insights, in terms of correlation and further hypothesis generation, but it is important to consider potential confounding factors when interpreting the results. While they offer a snapshot of exposure and outcomes, using longitudinal or more controlled study design can complement these findings and help strengthen the understanding of mycotoxin effects over time.

On the other hand, longitudinal studies allow the comparison of baseline data with future data, understanding changes and relationships between exposures and outcomes over time. Furthermore, evidence of causality and information regarding the strength of the association between mycotoxin exposure and adverse health outcomes can be obtained. Additionally, determining incidence rates and risks enables the identification of patterns in exposure and health outcomes within a population, supporting the identification of potential risk factors. Because of their position in the hierarchy of evidence, longitudinal studies are preferred within observational research designs.

Case-control studies involving mycotoxins are retrospectively identifying past exposure in patients who have developed a disease compared to controls who do not have the disease. To measure the association, odds ratios are used to check the proportion of an outcome in exposed individuals that can be attributed to a mycotoxin exposure. A systematic review of epidemiological studies by (Claeys et al. 2020) includes 13 case-control studies investigating the correlation between mycotoxin exposure and human cancer risk.

### Experimental studies

Ethical constraints and the often-impractical duration required to observe potential negative chronic health outcomes of mycotoxins may restrict the use of experimental study designs, such as randomized controlled trials (RCTs). It is not acceptable to conduct controlled intervention studies in which participants are deliberately exposed to high mycotoxin levels to assess adverse health effects. Ethical concerns can be mitigated by designing RCTs that ensure the experimental group receives an exposure level within the normal dietary range or at doses well below the tolerable daily intake (TDI). In the study of Phillips et al. (2020), causal linkage between mycotoxin exposure and child stunting was investigated by giving the experimental group a low-aflatoxin diet. In such studies, the experimental group would typically involve reduced mycotoxin exposure, while the control group reflects normal or background exposure levels, rather than administering higher doses. Human experimental studies in mycotoxin research primarily focus on toxicokinetic profiling, in which a very small, non-toxic dose is administered to investigate the absorption, distribution, metabolism, and excretion of a specific mycotoxin in the human body. However, given their high evidentiary power, RCTs remain valuable for establishing causal relationships following observational studies.

### Sample size

The sample size required to properly power the study depends on the study objectives (Levin 2005b). Prior knowledge of the effect size and the likely distribution of mycotoxins should be estimated by reference to the literature or pilot data. For example, in Sub-Saharan Africa similar villages in a small region may have 3 log variations in aflatoxin biomarkers (reviewed by Turner and Snyder 2021). In cohort studies, there is a need for sufficient participants to ensure that exposure categories to mycotoxins are well represented across different strata of covariates. The accuracy of the biomarker estimate should also be considered, as discussed for urinary FB1 measures. Oversampling by 5 to 10% is recommended to account for dropout and loss to follow-up and therefore maintain the statistical power in the study. For cohort studies over several years this oversampling should be increased. The study is sufficiently powered when the incidence rate of the outcome of interest in the exposed individuals is significantly different from the unexposed group (Kang 2021). Studies within mycotoxin research are typically investigating rare outcomes, low exposure frequencies, low effect sizes, etc., which is why they require a bigger sample size. When working with mycotoxins, accurately estimating the prevalence of a specific condition or outcome in advance is challenging. Investigators must rely on pilot data and assumed values for prevalence, significance level, and margin of error to guide their analysis. When the natural presence of some mycotoxins is low, working with large cohorts has the advantage of opening the possibility to detect rare outcomes, small or weak associations, account for variability in the population, and control for more confounders, often needed in mycotoxin research (Wang and Kattan 2020).

### Analysis and reporting of observational studies

Confounding can present a significant challenge in cross-sectional studies. While confounding is ideally addressed during the design phase, it is challenging in mycotoxin research due to the ethical and practical difficulties of conducting intervention studies that involve randomization, pairing, or matching. In an observational study, a (sub)population is observed, and claims are made based on the data. Fortunately, it is possible to control for confounders at the analysis phase using statistical techniques. Creating a “Table One” is a standard practice in observational studies to check if baseline characteristics are balanced. Confounding occurs when a variable is associated with the exposure of mycotoxins and influences the outcome, possibly resulting in a distortion of the association between exposure and outcome. One way to correct for confounding is stratification. Stratification involves analysing the association between exposure and outcome within levels of a confounding variable, followed by estimating an overall effect using weighted averages (Mantel and Haenszel 1959). However, this approach requires a large sample size and is limited when many confounders are present. In such cases, propensity scores offer a more scalable solution. These scores, estimated via logistic regression, represent the probability of exposure given selected characteristics (Heinze and Schemper 2002). Matching or weighting participants based on these scores helps balance baseline characteristics between exposed and unexposed groups, mimicking randomization. For example, individuals with similar dietary habits but differing mycotoxin exposure levels can be compared more fairly. This reduces bias and allows for more accurate estimation of exposure effects. These methods improve internal validity but may be applied differently depending on the study context. Many studies report non-random exposure to mycotoxins, which could be influenced by dietary habits, socioeconomic status, or geographical location (Abia et al. 2013; Gerding et al. 2014; Wallin et al. 2015; Martins et al. 2019). Within exposome research, isolating the pure effect of chronic mycotoxin exposure on health can be challenging. Moreover, it is impossible to carry out randomized or crossover designs. The primary mycotoxin exposure of interest is mixed up with some other, often unknown, factors associated with the outcome. To minimize these confounding factors, matching, restriction, and stratification at the design phase is advised. Matching could limit the sample size but allows pairing according to potential confounding variables. Restricting means limiting participation in the study to individuals who are similar (e.g. vegetarian) which can help control variability in exposure. However, when dietary patterns like vegetarianism are not independently associated with a health outcome of interest, variation in exposure due to diet is a relevant part of the causal pathway rather than a bias to be eliminated.

Assessing the relationship between mycotoxin biomarkers and health outcomes should be done while accounting for dietary and demographic confounders. Comparing health outcomes across groups with similar propensity scores but varying mycotoxin exposure levels is used to identify dose-response relationships in an exposure pattern analysis. These considerations should also be taken into account when estimating the impact of interventions (e.g., dietary guidelines or agricultural policies) aimed at reducing mycotoxin exposure.

### Study design and metadata

Optimal study design is key to both a powerful and reliable study. Design issues include study objective, availability of data, sampling methods, data collection, cost of the design, time implications, controls, and ethical considerations (Wang and Kattan 2020).

Robust sampling is essential for reliable biomonitoring outcomes. Inadequate methods can lead to bias, low response rates, and incomplete data, which may require unnecessarily large sample sizes. Leveraging existing secondary data during study design can improve efficiency and reduce costs (Carlson and Morrison 2009). Because specific mycotoxin exposures or related outcomes are often rare, even large cohort studies may yield limited data. For causal inference, appropriate internal controls such as unexposed individuals within the same cohort can strengthen comparisons (Wang and Kattan 2020). To ensure generalizability, studies should include representative samples of the target population and apply probabilistic sampling methods.

Mycotoxins are a diverse group of compounds, with significant variability in their physicochemical and toxicokinetic parameters across different mycotoxin groups. One notable consequence of this variability is the differing half-life within the human body. For this reason, it is important for HBM of mycotoxins that the sample collection is carried out consistently, at the same time of day, ideally following a standardized interval after the last meal. When this approach is infeasible, relevant information should be included in the metadata with the use of questionnaires and biological measurements to account for these variations.

Complete randomization is not feasible in observational studies investigating the effects of harmful substances, thereby limiting the analysis to the data available (Carlson and Morrison 2009). Therefore, selection bias can occur in epidemiological studies where subgroups are no longer completely representative of the overall population. If in mycotoxin research the exposed and unexposed groups differ in ways that predict the outcome, selection bias poses a problem for generalization. For instance, bias can occur if vegetarians are allocated to a certain study group while they are possibly more exposed to specific mycotoxins due to their higher consumption of plant-based products (Augustin Mihalache et al. 2024). Furthermore, it is recommended that investigators assess any systematic differences in outcomes and/or exposures between participants who completed the study and those who were lost to follow-up.

Improving standards for observational studies can be achieved by registering the study (Choi et al. 2017). Following reporting guidelines such as STROBE (STrengthening the Reporting of OBservational studies in Epidemiology) greatly improves the assessment of strengths and weaknesses of studies reported in literature, improves the quality of observational studies, and aids multi-centre international studies to adhere to local regulations and best practices (von Elm et al. 2007).

### Dietary intake records

Assessing dietary exposure to mycotoxins requires reliable and adaptable dietary assessment tools that accurately capture individuals’ food consumption patterns. Food Frequency Questionnaires (FFQ) are a suitable tool to routinely assess the frequency and quantity of food consumption for commodities at risk for mycotoxin contamination (Turner et al. 2008c). However, flexibility is recommended to accommodate new technology-based 24-hour dietary recalls or food diaries (Ocké et al. 2024). FFQs or food diaries tailored for mycotoxin research can improve interpretation of HBM data by linking reported food intake with mycotoxin occurrence in local food consumption databases (EFSA 2011; Heyndrickx et al. 2015; Seeuws 2017). Using standardized portion images enhances data accuracy, and combining FFQs with biomarkers strengthens exposure assessments of dietary interventions (De Nijs et al. 2016). Energy adjustments help generalize findings to the broader population (Willett and Stampfer 1986; Tomova et al. 2022). Although participants completing the FFQ are assumed to provide accurate and honest responses, it is often difficult for them to recall their dietary intake over extended periods, such as one month. In general, participants tend to recall healthy foods more easily than unhealthy foods. As a result, certain food items or nutrients could be underestimated. In addition, participants may alter their diet to simplify completing the questionnaire.

In large-scale studies, it is often assumed that dietary patterns and associated mycotoxin exposure are stable over time, as collecting 24-hour dietary recalls from all participants is logistically challenging. Moreover, HBM only provides a snapshot of the biological status of the participant, missing between and within day variability, which is crucial for short half-life mycotoxins. Additionally, bias may occur if participants inaccurately report their diet, avoiding mention of unhealthy foods or alcohol consumption. Standardizing and recording sample collection times enhances the rigor and reliability of study design. While FFQs are widely used, they have limitations specific to mycotoxin research. For example, key exposure sources like spices may be overlooked, as in studies in Bangladesh which found a high OTA and CIT exposure probably linked to local foods and/or spices, not staples (Kyei et al. 2022). Similarly, Penczynski et al. (2022) could not link DON exposure to specific foods, even with detailed FFQs. In addition, specific mycotoxin-related parameters can be incorporated or added into the questionnaire to enhance their relevance.

Another approach for future studies is the use of food intake biomarkers instead of FFQ. These are compounds or their metabolites that are characteristic for certain food items and can be analysed in physiological samples after food intake. For example, it was shown in a recent study that urinary metabolites of alkylresorcinols are potential biomarkers for grain intake (Frank et al. 2025). Depending on cohort sample availability, alternative matrices, such as human stool, could also be utilized for dietary intake estimation in parallel (Diener et al. 2025). These could be promising approaches for future studies as especially urine samples are available from many human intervention studies allowing simultaneous mycotoxin and food intake HBM. This approach allows to overcome the biases commonly associated with FFQs, dietary recalls, and food records, where participant reporting is often inaccurate.

## Matrices, sampling, clean-up and mass spectrometric analysis

### Matrices and sampling

In general, several matrices such as urine, blood (including plasma, serum) or breast milk have been used in the past for HBM of mycotoxins. However, the choice of matrix, sampling method, and sampling conditions must align with the desired outcomes and the analyte(s) of interest. For example, in blood samples only OTA, 2’R-OTA, AFB1-Lys, CIT, and EnB, are currently of relevance as biomarker or potential biomarker (see Sect. Biomarkers). OTA is strongly binding to serum albumin resulting in a half-life in human blood of 35 days after oral ingestion (Studer-Rohr et al. 2000). For this reason, it represents one of the most detected mycotoxins in HBM studies using blood samples (De Ruyck et al. 2020; Warensjö Lemming et al. 2020; Bastos-Moreira et al. 2023, 2024a). OTA is even detectable in all blood samples under low exposure scenarios (Warensjö Lemming et al. 2020). Under high and chronic exposure, such as in a cohort of 436 pregnant women in rural Bangladesh, OTA was the most frequently occurring mycotoxin and was also detected in 95% of urine samples without specific analyte enrichment (Kyei et al. 2023).

As venous blood sampling is invasive, several minimally invasive microsampling methods and porous storage supports have been developed, enabling home or remote sampling as an alternative to the traditional venipuncture. This approach reduces the burden on participants and the logistical challenges associated with sample withdrawal and storage, thereby facilitating the collection of a greater number of samples. Consequently, the possibility to perform large-scale HBM studies with a large number of participants or serial samplings is opened (Sueck et al. 2020; Bastos-Moreira et al. 2023). The first micro-sampling method was the use of DBS for the analysis of OTA (Cramer et al. 2015). Usually, 100 µL or 1–3 drops of blood, obtained by a simple finger prick with a lancet, are spotted on cellulose-based filter paper cards, air-dried, punched or cut out, extracted and analysed by HPLC-MS/MS. It was shown that basic parameters such as spotting volume and hematocrit had not much influence on the results, allowing quantitative measurements (Osteresch et al. 2016). As follow-up, a DBS and dried serum spot (DSS) method was developed and validated for 27 mycotoxins and metabolites (Osteresch et al. 2017). For most analytes, LOQs were in the lower pg/mL range and excellent recovery rates were achieved using matrix-matched calibration. Currently only OTA, 2’R-OTA, CIT and EnB are of relevance as (potential) biomarker in hematic matrices but recent findings of multiple mycotoxins and mycotoxin metabolites in plasma indicate a future potential for HBM, when no urine samples are available (Tesfamariam et al. 2022; Ning et al. 2024). Furthermore, in large cohort studies, in which numerous parameters are analysed from a limited amount of blood, DBS offer the great advantage of requiring 100 µL or less blood for HBM of OTA and the abovementioned mycotoxins.

Besides DBS, another micro-sampling technique is volumetric tip microsampling which is currently commercially available as volumetric absorptive microsampling (VAMS) Mitra^®^ tips (Trajan Scientific and Medical, Torrance, USA), and as Tasso + or Tasso-M20 (Tasso, Seattle, USA). These methods involve collecting a small volume of blood through a skin prick. VAMS Mitra^®^ tips utilize polymeric absorbent tips to collect a fixed volume of blood, which is then air-dried. VAMS Mitra^®^ tips offer hematocrit-independent precision for quantitative mycotoxins analysis (Stove et al. 2012; De Kesel et al. 2015; Vidal et al. 2021). Tasso +^®^ is an innovative device that adheres to the skin and creates a vacuum to draw blood from capillaries after a lancet puncture. A key innovation of the Tasso +^®^ device lies in its compatibility with various collection containers, enabling users to autonomously collect both fixed volumes of dried blood on cellulose supports (Tasso-M20) and liquid haematic samples in tubes (Tasso+^®^), enhancing flexibility and sample volume. At present, the most significant disadvantage of VAMS Mitra^®^ tips remains, besides costs, the low sample volume (max. 30 µl), limiting the mycotoxin detection capabilities. Nevertheless, VAMS Mitra^®^ tips have proven to be very practical in the MISAME-III trials in which multi-mycotoxin exposure assessments were performed on a large-scale longitudinal study in Burkina Faso (Tesfamariam et al. 2022; Bastos-Moreira et al. 2023, 2024a, 2024b). Tasso +^®^ also has a relatively high cost compared to DBS sampling; however, it offers the advantage of compatibility with traditional collection tubes and allows for the collection of a larger sample volume (up to 600 µL), which is beneficial for biomedical analyses (Jacobson et al. 2024; Tasso Inc. 2025). For any blood sampling it is important to record the time since the last meal, as fasting can significantly influence the analysed mycotoxin biomarkers.

Studies on mycotoxin exposure are usually conducted as part of larger studies which often include a comprehensive description of the state of health of the participants. In this context, blood and serum samples are commonly collected to determine infections, nutrient levels, or haemoglobin concentration (Humphrey et al. 2015). In this case, the sampling itself is not the most important part for mycotoxin analysis. Instead, the bigger challenge is keeping the sample stable during storage and worldwide shipping to qualified laboratories. Here, dried blood samples such as DBS or VAMS Mitra^®^ can also be useful. The stability of mycotoxins in blood spots dried on filter paper cards has been studied. Usually, these cards are handled and shipped at room temperature, which might be a crucial factor. Indeed, storing the filter cards at room temperature led to a decrease in recovery, but after one week, recoveries were still above 61%. Storage between 4 and − 18 °C is recommended as all recovery rates remained above 76 and 88% after 24 weeks respectively (Osteresch et al. 2017). Due to this benefit, DBS has recently been used in large cohorts, e.g. in Swedish adolescents (*n* = 1105) (Warensjö Lemming et al. 2020). The stability of mycotoxins using volumetric absorptive microsampling revealed similar results. Storage of VAMS at 4 °C or room temperature revealed acceptable recovery rates (Vidal et al. 2021).

Despite the analytical improvements for hematic matrices, urine samples are the main matrix used for HBM of mycotoxins. This matrix has been applied since the earliest studies on mycotoxin exposure and was also used in the first multiple mycotoxin analyses, including those conducted in Belgium (BIOMYCO study), Germany, Haiti, and Bangladesh (Heyndrickx et al. 2015; Gerding et al. 2015). The great advantage is that urine collection is easy and non-invasive, allowing the collection of samples from large cohorts. Furthermore, quantitative data obtained from urine samples can be used to calculate the provisional mean daily intake if toxicokinetic data are available (see Sects. Biomarker and Recent trends and challenges in interpretation of human biomonitoring data). Urine samples can be collected as 24-hour urine or spot urine, taken at any point in time during the day, or as first-morning urine. The gold-standard for urine sampling is 24-hour urine collection as it allows for the calculation of the total excretion of a compound within this period. Nonetheless, this approach poses significant challenges related to collection, transport, and handling, making it generally impractical for large cohort studies. When 24-hour urine is not available due to these limitations, first-morning void collection is often preferred over spot urine as it is more concentrated which reduces intra-day variability. However, spot urine samples are in general of limited value for calculating the average daily excretion, particularly for compounds with half-lives of a few hours (Warth et al. 2013a). If spot urine samples are collected the time since the last meal should be recorded. In a recent study, 24-hour urine was collected from 72 participants to compare mycotoxin exposure between omnivores and vegans (Penczynski et al. 2022). DON glucuronide was quantified in 57% of the urine samples without any significant difference between vegans and omnivores. This is comparable with other studies collecting spot urine samples in Germany. Gerding found 54% and 57% quantifiable urine samples in two other German cohorts (Gerding et al. 2014, 2015). However, in the BIOMYCO study in Belgium, DON-15-glucuronide was the main urinary DON biomarker and was found in all urine samples from the 239 adults and 155 children, within the ng/mL range (Heyndrickx et al. 2015). The only significant difference in the vegan study was observed for serum OTA, with levels being 2-fold higher in vegans compared to omnivores (Penczynski et al. 2022).

A challenge in using urine samples is the potential high variability due to dilution, which is influenced by the individual hydration status and kidney function. To compensate for this, several normalization methods can be applied, including adjustments based on creatinine levels, specific gravity, conductivity, osmolality, or collecting 24-hour urine (Lermen et al. 2019). Most studies use creatinine to normalize spot urine samples, which is the dehydration product of creatine and creatine phosphate. It was estimated that around 1.5–2.5% of human creatine is converted to creatinine (Crim et al. 1975). The renal excretion of creatinine is regarded as relatively constant over the day in healthy individuals, allowing to calculate metabolite/creatinine ratios, typically presented as µg metabolite per gram of creatinine (µg/g creatinine) (Johner et al. 2015). However, a recent HBM study clearly showed that creatinine levels are biased when meat and fish are consumed as these are exogenous sources of creatinine. Abraham et al. obtained 26% lower creatinine-adjusted levels of 2,3-dihydroxpropylmercapturic acid, a diet-independent endogenous C3-metabolite, in omnivores compared to vegans while the excretion per 24 h was similar in both groups (Abraham et al. 2023). Another limiting factor is the age dependence of creatinine excretion, making it challenging to compare creatinine-corrected HBM values between children and adults (Kestenbaum et al. 2022). Further limitations of creatinine and influencing factors can be found in the literature (Carmine 2025). Because of these reasons, creatinine-adjusted values might be good on longitudinal individual level, but may introduce errors when adjusting data on population level because of the inter-individual variability (Kestenbaum et al. 2022). Other adjustment methods less affected by inter-individual variability introduced by muscle mass, diet, health and physical activity, like specific gravity or osmolality, are preferred to adjust for urinary dilutions in observational studies targeting a population. In particular, specific gravity tends to work better in highly diluted samples as it is less influenced by metabolic activity, individual variability, kidney function, age and low-muscle mass individuals. Since it is more consistent across populations, specific gravity is often the preferred normalization method for urinary dilution outside the field of mycotoxin research (Suwazono et al. 2008; Sauvé et al. 2015; Kestenbaum et al. 2022).

In addition to report the normalized results (e.g. in µg/g or ng/mg creatinine), it is recommended to provide the data also in µg/mL or ng/mL urine to enable the comparison between studies. Moreover, since the exposure is inherently dynamic, measurement of spot samples is prone to bias depending on the specific day of assessment. As discussed in Sect. Study design and dietary intake records, evaluating long-term exposure at the population level requires more robust approaches, such as repeated cross-sectional assessments or tools specifically designed for longitudinal evaluation of individual dietary exposure. FFQs, for instance, can partially address this need by providing insights into habitual dietary patterns over time. In future mycotoxin HBM studies, alternative urinary correction methods, such as specific gravity, may provide more reliable and consistent results. However, creatinine correction remains widely used, as it facilitates comparison with previously conducted studies in the field.

Besides blood and urine, breast milk is another alternative matrix for HBM studies. Mycotoxin levels measured in human breast milk reflect maternal intake of such food contaminants and allow to calculate exposure of infants, as observed in the first longitudinal study with OTA (Muñoz et al. 2014) and in studies on other mycotoxins (Cantú-Cornelio et al. 2016; Warth et al. 2016a; Braun et al. 2020; Ayeni et al. 2024). Human milk samples should be collected at various time points during the breast-feeding period since the lactational transfer from maternal blood into breast milk can differ, leading to higher levels in colostrum than mature milk. For OTA, the highest milk/plasma (M/P) ratio (0.40 ± 0.26) was found in the first days of nursing and declined thereafter to lower M/P ratios (0.15 ± 0.26) at later stages (Muñoz et al. 2014). For other mycotoxins, such data is not available as it would require parallel sampling of maternal blood and breast milk. Multi-mycotoxin analysis of human milk samples from Nigerian mothers revealed occurrence of AFM1, AME, BEA, CIT and DH-CIT, EnB, EnB1, OTA and STG (Braun et al. 2022; Ayeni et al. 2024). FB1 was detected in milk from Tanzanian mothers (Magoha et al. 2014); however, as several extremely high values were reported this might be due to the instability of used standards in methanol (Turner and Snyder 2021).

As alternative sampling technique, hair analysis, which is routinely applied in forensic and clinical toxicology (Kintz 2018), was recently applied in a large-scale pilot study to depict chronic exposure to mycotoxins confirming the accumulation of aflatoxins, T-2 toxin, and enniatins in hair matrix (Narváez et al. 2022). However, although the analysis of hair represents an established tool in toxicology to measure chronic exposure, its application in HBM of mycotoxins is still at an early stage. Analytical guidance values and standardized procedures for these analytes in hair have to be established, and the contribution of several variability factors must still be clarified.

Stool sample analysis for general HBM studies is typically not conducted, in part because it does not necessarily provide a marker of systemic bioavailability. This is particularly important to mycotoxins such as fumonisins with very low and high interindividual variation in bioavailability (van der Westhuizen et al. 2011; Torres et al. 2014). However, in limited cases stool samples especially of infants have also been used for HMB of mycotoxins. Stool samples have the great advantage that sampling is non-invasive. Krausová et al. (2022) developed an LC-MS/MS method for the analysis of more than 30 mycotoxins and metabolites in infant stool samples. For sample preparation of stool samples a rather simple ‘dilute, filter, and shoot’ approach was used (Krausová et al. 2022). In a recent study by Ayeni et al. (2024) stool samples of 82 infants were analysed for mycotoxins by this method. Furthermore, the composition of the microbiota was characterized by 16 S rRNA gene amplicon sequencing and correlated with mycotoxin patterns in the stool samples. Although the sample size was rather small, the results indicate that mycotoxins might influence the gut microbiota (Ayeni et al. 2024).

### Sample clean-up

#### Urine

Several protocols for clean-up and sample preparation have been developed in order to improve sensitivity of analytical methods and to remove interferences from the biological matrix. For urine samples, solid phase extraction (SPE), liquid-liquid extraction, as well as immuno-affinity chromatography are routinely used (Njumbe Ediage et al. 2012; Warth et al. 2013b; Cramer and Humpf 2017; Arroyo-Manzanares et al. 2021). The use of immunoaffinity chromatography (IAC) columns in particular enables effective separation of matrix components and specific enrichment of the target analytes (Solfrizzo et al. 2011). However, due to their high selectivity, these antibody-based methods are only suitable for a limited range of analytes and are associated with high costs. Furthermore, the binding of phase I and phase II metabolites is often limited and needs to be verified. Often, treatment with glucuronidase/sulfatase is required to facilitate binding of the latter. The number of IAC applications for the purpose of large-scale multi-mycotoxin analysis is therefore limited and mostly applied to small cohorts (Arroyo-Manzanares et al. 2021). One of the first multi-mycotoxin methods for human urine samples based on IAC was published by Ahn et al. (2010). In this study, AFM1, FB1, FB2 and OTA were analysed using three separate IAC columns. After combining the eluates, analysis was performed by HPLC-MS/MS in a single chromatographic run. By using a multi-IAC column (6-in-1), Rubert et al. (2011) were able to simultaneously determine eleven mycotoxins and mycotoxin metabolites, namely the aflatoxins AFB1, AFB2, AFG1 and AFG2, DON, T-2, HT-2, FB1, FB2, OTA, and ZEN. Solfrizzo et al. (2011) combined the multi-IAC column with SPE and integrated an enzymatic hydrolysis, so that in addition to AFM1, DON, DOM-1, FB1, OTA, α-ZEL and β-ZEL, further phase II metabolites could be indirectly detected.

SPE methods based on hydrophobic interactions also have limitations, especially for metabolites with low retention. Polar compounds, in particular glucuronic acid conjugates, are often not sufficiently retained by SPE materials and for this reason overall sample preparation for multi-mycotoxin analysis is often characterised by compromises (Warth et al. 2012a, 2013b; Arroyo-Manzanares et al. 2021). To date, Šarkanj et al. 2018 achieved the lowest detection limits in a multi-method by an enzymatic hydrolysis of glucuronides followed by SPE clean-up. An extension of SPE is the use of online SPE, where the extraction column is part of the (U)HPLC-system and via valves connected to the main separation column(s). Besides the reduction of the need for manual sample handling, this technique increases time efficiency, reduces solvent consumption, and is ’greener’ due to the application of reusable extraction columns. Additionally, the high level of automatization allows a minimization of analyte loss and degradation and can improve accuracy and precision (Rodriguez-Mozaz et al. 2007; Pan et al. 2014). An online SPE-UHPLC-MS/MS method was developed for the analysis of 11 mycotoxins and metabolites in urine samples by Schmidt et al. (2021b) and was recently further extended to 36 mycotoxins and bio-measures (Kuhn et al. 2025). Liquid-liquid extraction, either with acetonitrile based on QuEChERS (Quick, Easy, Cheap, Effective, Rugged, Safe) protocols or by salting-out assisted extraction with ethyl acetate are other commonly used sample preparation techniques used also for large cohorts (Njumbe Ediage et al. 2012; De Santis et al. 2017; Vidal et al. 2018a, 2018b; Martins et al. 2019; De Ruyck et al. 2020). Simple evaporation of the volatile organic phase allows concentration of analytes, but matrix removal is limited, and the extent of phase separation needs to be evaluated carefully for reliable quantification.

Alternatives to these clean-up methods are the application of dilute-and-shoot (DaS) approaches, in which urine samples are directly analysed by HPLC-MS/MS after dilution with aqueous solvents and centrifugation. This method offers two key advantages: the ability to integrate structurally diverse analytes and high time efficiency. However, this approach eliminates the separation of matrix components and the potential concentration of analytes, which usually results in higher detection limits. Although the sample preparation is non-specific, the method’s extension still depends on the compatibility of the analytes with the liquid chromatography and mass spectrometry parameters used (Warth et al. 2013b; Cramer and Humpf 2017; Arroyo-Manzanares et al. 2021). DaS methods for the analysis of 15 and 23 mycotoxins and mycotoxin metabolites were developed by (Warth et al. 2012a) and (Gerding et al. 2014, 2015). These methods were later extended by additional mycotoxins and metabolites and extensively used with large cohorts in the past (Warensjö Lemming et al. 2020; Kyei et al. 2023).

As an alternative on-site sampling and clean-up strategy, a dried urine spot (DUS) method for 14 relevant mycotoxins and metabolites was developed. Urine samples are treated with glucuronidase, spotted on filter paper strips, extracted and analysed by HPLC-MS/MS using isotopically labelled standards. Stability experiments showed that the DUS can be stored at room temperature for up to 28 days without a major decrease in the analyte concentrations. The sampling protocol required minimal laboratory equipment and could be easily performed on-site in rural areas (Schmidt et al. 2021a).

#### Blood

For the analysis of mycotoxins in plasma, serum or blood samples several methods for sample preparation have been developed and routinely used in the past. These include protein precipitation, liquid-liquid extraction, SPE, QuEChERS based methods as well as IAC clean-up followed by HPLC with fluorescence detection (FLD), HPLC-MS/MS or enzyme linked immunosorbent assay (ELISA) as detection techniques (Arce-López et al. 2020). These methods are routinely used in HBM as they provide low detection limits and high recovery rates. However, as already described above microsampling techniques such as DBS or volumetric microsampling became more and more into the focus as these techniques need only minimal sample preparation (Jacobson et al. 2024). For DBS and VAMS just a simple extraction step with organic solvent mixtures is needed prior to HPLC-MS/MS analysis but, depending on the blood volume sampled, lower analytical performance has to be taken into account (Osteresch et al. 2017; Vidal et al. 2021).

### Mass spectrometric analysis

For mycotoxin detection in multi-analyte methods, mass spectrometers with electrospray ionization (ESI) are almost exclusively used (Warth et al. 2013b; Arroyo-Manzanares et al. 2021). This technique offers the best available ionization efficiency but is also prone to signal suppression or enhancement (SSE) due to coeluting matrix components (Matuszewski et al. 2003; Stokvis et al. 2005; Schuhmacher et al. 2008). Since this SSE is associated with a loss of sensitivity and accuracy, the above-mentioned sample preparation methods describe compromises between the minimization of these matrix effects by removing matrix components during sample clean-up, and the ability to detect a broad spectrum of analytes with reasonable sample preparation efforts and adequate sensitivity. Nevertheless, SSE may remain the most critical parameter affecting accurate quantification, as variables such as individual water uptake, nutrition, sex, age, health, and socio-economic status can affect the biological matrix. Therefore, the use of stable isotope-labelled standards, whenever possible, is recommended to correct for matrix effects and achieve more accurate, precise, and reliable quantification.

#### Tandem mass spectrometry (MS/MS)

Reliable identification and quantification of mycotoxins and their metabolites demand high levels of selectivity of the mass spectrometric methods. In the beginning of HPLC-MS/MS analysis of mycotoxin biomarkers, these criteria were not defined and a lot of the ground-breaking research on AFB1 exposure using AFB1-Lys as biomarker was probably only possible because the single MRM transition *m*/*z* 457.3–394.2 was regarded as sufficient for identification (McCoy et al. 2005).

Nowadays, two MRM transitions with a defined intensity ratio are the standard in multi-mycotoxin methods but unfortunately this is no guarantee for sufficient selectivity (European Commission 2024). Therefore, special attention should be given to uncommon detects differing from previous results such as higher incidences of OTα compared to OTA in urine or blood samples, frequent detects of DON or other rapidly eliminated metabolites in blood samples, detects of AFB2 without AFB1/AFM1 or T-2 without HT-2. However, this does not mean that the data reported in these publications is false or incorrect, but the selection of additional or more specific MRM transitions can strengthen such findings.

#### High-resolution mass spectrometry (HRMS)

In addition to MS/MS, high-resolution mass spectrometry (HRMS) is becoming increasingly important. Due to its high resolution and additional selectivity, HRMS facilitates the analysis of complex mixtures. Non-targeted analysis allows the screening of previously unknown compounds and enables retrospective data analysis. However, the scope of HRMS detection lies primarily in qualitative analysis. Tandem mass spectrometers are more commonly used for quantitative analysis, as they often prove to be superior to HRMS devices in terms of sensitivity. However, ongoing technical developments in HRMS combined with powerful data acquisition and processing may enable qualitative and quantitative analysis in the future (Vidal et al. 2018b; Arroyo-Manzanares et al. 2021; Lindemann et al. 2022). So far, HRMS-based methods for HBM of mycotoxins have been used only sporadically but the call for comprehensive multi-analyte methods seems to promote their use in this field (Debegnach et al. 2020; Ndaw et al. 2021; Carballo et al. 2021; Narváez et al. 2021). Critical confirmation of possible detections is also of high importance for HRMS measurements. While the application of two MRM transitions is common practice for unit resolution HPLC-MS/MS systems, confirmation of detects by recording of HRMS fragments is not always implemented and may lead to low specificity (Dasí-Navarro et al. 2022). Special attention should also be given to the reporting of the applied mass spectrometric parameters and especially to criteria for data processing such as mass extraction window, and the accepted range of mass error.

### HPLC with UV or fluorescence detection

HPLC with UV or fluorescence detection was besides thin layer chromatography (TLC) the key technique in the first years of HBM. UV-active and fluorescent analytes like AFs, OTA and CIT could be detected after analyte-specific purification procedures (Orti et al. 1986; Gareis et al. 1988; Castegnaro et al. 1990). For other non or little UV-active compounds like FB1, approaches applying derivatization steps are published (Shetty and Bhat 1998). Advantages of these methods are that no matrix or matrix matched calibration or expensive stable isotope-labelled standards are required. Furthermore, they can also be more easily operated than MS in regions with unreliable power supply. Due to low sensitivity, UV detection is no longer used in current detection methods while fluorescence detection of OTA and AFM1 in combination with immunoaffinity purification still remains an important tool for HBM (Redzwan et al. 2015).

### ELISA

Although ELISA is capable of quick and sensitive analysis, its use in HBM is limited due to possible cross-reactivity with target metabolites or matrix components which may lead to less reliable quantification. Studies using ELISA are strongly encouraged to conduct additional physico-chemical analysis of a subset of perhaps 20% of samples; this could include HPLC-MS/MS approaches. Similar to IAC, the need for specific antibodies reduces the range of analytes, making this technique unsuitable for multi-mycotoxin analysis (Arce-López et al. 2020). Three major uses of ELISA have occurred in HBM research. Firstly, the measurement of AFM1 in urine (Ali et al. 2017a), for which kits are commercially available. Secondly, a highly specific antibody was produced against AF-Alb that has created an extensive data set, based on its use in an in-house ELISA (Wild et al. 1990), on chronic AF exposure, predominantly in Sub-Saharan Africa (Turner and Snyder 2021). Importantly strong dose response correlations are reported when comparing ELISA data with HPLC-MS/MS and HPLC-FLD data (Wild et al. 1990; Scholl et al. 2006). Thirdly, ELISA has been applied widely for the analysis of OTA in blood (plasma/serum) and results obtained by ELISA and by HPLC showed a satisfactory correlation (Märtlbauer et al. 2009).

### Analytical data interpretation and evaluation

The development of methods with improved sensitivity – meaning lower LOD and LOQ values can help to improve the quality of exposure assessment and minimize the number of left-censored data points. Thus, requests for lower LODs and LOQs are well justified, and the evaluation of analytical methods strongly depends on these values. Accordingly, in the case of sample sets with left-censored data, it is recommended to critically evaluate the validated concentration range on the basis of the reported LOQ values and the lowest reported analyte concentrations in the sample set. Assuming a truncated normal or lognormal distribution, large gaps between the lowest reported concentrations and the LOQ may indicate the need for additional validation experiments at the low concentration range (Pleil et al. 2014). Extending the analytical methodology to the low concentration range, however, might also require a re-evaluation of the biomarker validation data for these levels.

## Recent trends and challenges in interpretation of human biomonitoring data

In recent decades, there has been a notable rise in studies that analyse mycotoxins and their metabolites in blood plasma or serum, urine, and breast milk for investigating human exposure. Along with dedicated methods for analysis of biomarkers of exposure to distinct mycotoxins, multi-mycotoxin methods are increasingly applied for the simultaneous determination of multiple analytes as described in the previous chapter. This development in HBM has led to a wealth of monitoring data obtained in different settings to facilitate exposure assessment for use in epidemiology, exploring as yet ’understudied’ emerging mycotoxins, and assessing the efficacy of interventions to mitigate critical exposures. Yet, the available monitoring data for mycotoxins require a careful interpretation when assessing the biomarker-based estimates of intake and then exposure related risks.

### Descriptive and risk-based approaches in HBM data interpretation

The two main options for interpreting biomonitoring results are descriptive and risk-based approaches (National Research Council 2006). The starting point for both is a statistical analysis of the data, typically in the form of a data distribution (with mean, median, percentiles) for the mycotoxin biomarker concentrations in a given matrix and study group.

In a *descriptive approach*, the data distribution will serve to establish a range of HBM values within which individual or subgroup results can be compared and inform who is most exposed and who least. Depending on the biomonitoring setting, this may pertain to different age groups, diseased and healthy persons or to other characteristics including geographical location, climate and dietary habits of the study population. For instance, aflatoxin biomarker prevalence and concentrations in persons of Sub-Saharan countries are far higher than those found in European populations with access to safer food (Routledge et al. 2014; Chen et al. 2018; Schrenk et al. 2020a; Smith et al. 2022). Biomarker results may be further used by examining correlations with FFQ data and food items as the source of mycotoxin intake (see Sect. Study design and dietary intake records). Furthermore, comparing biomarker levels in occupationally exposed groups to ’controls’ with dietary contaminant intake alone can reveal additional workplace-related exposures. In short, HBM data provide first insights on prevalence for both dietary and occupational exposures combined.

Reference values in human biological fluids (e.g. blood, urine) are derived by statistical methods from surveys and repeated measures of chemicals, and further used by the German HBM Commission to define concentrations (HBM-I and HBM-II) with no increased risk for adverse health effects or with the need for intervention to reduce an exposure (Umwelt Bundesamt, 2023). The European Human Biomonitoring Initiative (HBM4EU) - aimed to further establish HBM as important tool for determining population exposure to chemicals and as part of health-related risk assessments - agreed on a strategy for deriving Human Biomonitoring Guidance Values (HBM-GVs) (Louro et al. 2019; Apel et al. 2023). These HBM-GVs are guidance values that correspond to internal exposure levels at which there is no appreciable health risk, and thus a valuable reference for a comparison with biomarker concentrations found in individuals and population groups. Regarding mycotoxins, an HBM-GV is presently only available for DON (23 µg/L urine for total DON, at the group TDI of 1 µg/kg bw; Mengelers et al. 2024). It has served to assess total urinary DON levels of 1270 adult participants from six European countries (Namorado et al. 2024), along with the common approach of converting biomarker results to exposure estimates for a risk assessment (see Sect. below).

The *risk-based approach* for evaluating newly obtained HBM data is challenging and requires information on relationships between biomarker concentrations and mycotoxin intake from human and/or animal studies or toxicokinetic modelling, as well as toxicity profiles including mode of action. But this kind of information is often not yet available for many emerging mycotoxins (e.g. *Alternaria* toxins, BEA, enniatins, moniliformin).

Only for a few contaminants, i.e. for AFB1, lead or mercury, biomarker dose-response relationships were developed in epidemiologic studies. These data-rich contaminants are amenable to a *direct* risk analysis by determining where on the exposure-response curve a person is.

### Exposure evaluation

The common approach to evaluate the exposure using HBM data is to convert biomarker measurements into human exposure. This requires information on the fraction of an ingested amount of mycotoxin that is transferred to urine within a day or present in blood. Such values – compiled in Table 11 – were obtained in studies with numerous human volunteers exposed to known amounts of a given mycotoxin using food measurements, followed by collection of their body fluids for analysis of parent compound and key metabolites (see Sect. Biomarkers). Several studies involved only a few adult individuals who received single doses as pure compound or in a food matrix. Some caution is indicated when transfer rates are applied to calculate the probable daily intake (PDI) of mycotoxins (for calculation of PDI see Gerding et al. 2015). Unless specifically tested, transfer rates may vary in children, especially the very young, or additionally for subgroups with diseases including liver and/or reduced renal function. For OTA, the Klaassen equation has been used to estimate the continuous exposure to OTA based on blood plasma concentrations, and assumptions for plasma clearance and bioavailability (details in Duarte et al. 2011).

In addition, differences in phase I and phase II enzyme activities may contribute to variation in the percentage transferred to urine or observed in blood. Thus, most estimates of exposure are based on average transfer rates for a population. It remains critical to explore the toxicokinetic for all mycotoxin biomarkers that will be used to estimate dose, including the relationship between mycotoxin ingested and the percent transfer to urine or blood and to be aware of the limitations and variance of that estimate. For example, for DON or AFM1 in urine the variations are relatively small, compared to FB1, where any given biomarker measure could reflect a 9-fold variation in the intake estimate for any given individual (see Table 11). For some mycotoxins only animal data is available with a potential for variance in transfer rates compared to humans and/or variance related to high doses being used in animal studies that can additionally modify the kinetics. Thus, the source of data for transfer rates and its limitations also have to be considered.

Further pieces of information are required to use the biological measurement of toxin/metabolite in a sample to estimate exposure or intake. Firstly, individual bodyweight is required and typically this is quite accurate. When not available many studies use average bodyweights relevant by country and age group. This can be valuable as estimates within large cohorts – but likely less reliable to understand the true distribution of data sets. Potentially, this can lead to misinformed interpretation if attempts to link these estimates of intake to a biological response or health effect are made. Thus, it is recommended to obtain bodyweight alongside collection of the bio-measures. For children collecting both weight and height data can be useful where growth is of interest. For studies on infant growth in developing world regions, ensuring the accuracy of age can be a challenge in some settings.

When 24-hour urine collection occurs, the total volume is important to record. If this is not noted, and for collection of first morning voids or spot urine then estimates of typical daily urine output is used – often between 1 L and 2 L for adults (Manz et al. 2012) and 0.65 to 1.16 for children (EFSA 2014b). Again, this may lead to imprecise estimates of intake at the individual level but is useful at the cohort level. Where blood-based biomarkers are used then assumptions are blood volume and in the case of albumin bound biomarkers the albumin concentration may be relevant. However, in studies in Benin and Togo, nutritional status did not seem to influence AF-Alb levels (Gong et al. 2004). When using longer-term biomarkers such as AF-Alb/AFB1-Lys, the reported transfer is based on assuming exposure occurring repetitively on a daily basis ahead of the measurement. Thus, in chronically exposed populations any given measure represents an integrated estimate of 2–3 months exposure, which can be valuable to smooth out the potential variance in daily exposure and report “typical” exposure. However, any given measurement may be alternatively derived from days of limited exposure and occasionally high acute exposure. Of course, the interpretation of any health impact may be very different say for an infant receiving 90 days’ worth of AFB1 in 1 day (a very large acute exposure) compared 1/90th of that amount evenly spread over 90 days, perhaps modest chronic exposure.

### HBM-based risk assessment

To put biomonitoring results in a risk context, biomarker-based exposure estimates are compared with HBGVs or used to calculate a MoE. As outlined in the introduction, international or national food safety authorities assess toxicity data available from animal experiments and mode of action information for some major mycotoxins to derive TDI values or advice to apply a MoE approach to assess risks for carcinogenic and genotoxic mycotoxins. For contaminants with a poor toxicological database, e.g. *Alternaria* toxins, the threshold of toxicological concern (TTC) can be used to define an external exposure which causes no harm. Yet, for numerous compounds, including emerging mycotoxins, no HBGV value could be derived so far, a situation which hampers an assessment of a potential risk for such compounds when detected in HBM studies.

Several uncertainties in biomarker-based exposure estimates were discussed above. As further illustrated in two recent articles, urinary excretion rates applied to a given biomarker level will affect the calculated exposure (PDI). In a study from Hungary (Szabó-Fodor et al. 2022), the authors calculated PDI values for DON, FB1, OTA and ZEN for their study group using either mean urinary excretion rates for piglets or for humans and then compared the PDI estimates to TDI values: the lower urinary excretion rate for FB1 in humans than piglets (0.5 vs. 2.6%) resulted in a higher percentage of samples which exceeded the TDI. An impact on DON and ZEN exposure estimates was small (0 or 5% exceedance of the TDI) whilst PDI estimates for OTA exceeded the (outdated) TDI in all samples. Of note, OTA levels in urine of healthy people were higher than those of coeliac patients, and in both groups higher than previously reported data for healthy Hungarians (Fazekas et al. 2005).

One study presents a useful approach to risk assessment and involved biomonitoring of 36 pregnant Dutch women for several mycotoxins (McKeon et al. 2024). To assess the risk of total DON measured in 24-hour urine two approaches were chosen by the authors, one comparing DON urine levels to an HBM-GV of 23 µg/L derived within the HBM4EU Initiative (Mengelers et al. 2024), the other using reverse dosimetry to convert urine concentrations to estimated daily intake and relate it to the group TDI of 1 µg/kg bw/day derived for DON. Both approaches result in similar distributions with low hazard quotients (HQ all < 1), indicating that no adverse health effects were expected. In the same study, urine concentrations for ZEN and its metabolites were used to calculate intakes for each participant by reverse dosimetry, applying a low and a high-end urinary excretion rate (9.4 or 36.8%) for a ’worst’ and ’best’ case scenario. Whilst this ’exercise’ resulted in different distributions, the overall outcome was comparable, with HQs clearly below 1, indicating that the level of exposure in this group was of no concern (McKeon et al. 2024). For OTA levels measured in all serum samples from these women, the authors chose to assess related risks by calculating intake based on the parameters of a human study (Studer-Rohr et al. 2000) and comparing the range of exposures to BMDL_10_ values derived by EFSA (Schrenk et al. 2020b) for non-neoplastic (nephrotoxic) and for neoplastic (kidney tumors) effects. Except for one outlier, all HQ values (for the respective MoEs) were well below a level of concern. These two studies, although based on a rather small number of participants, are examples for a careful interpretation of biomarker data, as the authors did not just rely on point estimates but applied several scenarios to assess the exposure-related risks.

A recent HBM study analysed urine biomarkers of CIT exposure in Germany, also since data on CIT occurrence and levels in food commodities are scarce (Degen et al. 2023). The results for children and adults revealed widespread exposure to this nephrotoxic mycotoxin and higher biomarker urine levels in children than in adults. Biomarker concentrations in spot urine were used to calculate the ranges of CIT intake, based on age-adjusted daily urine volumes, individuals’ body weights, and a mean urinary excretion rate determined previously in adult humans (Table 11). When CIT intake estimates were compared to an amount of 0.2 µg/kg bw per day defined as ’level of no concern for nephrotoxicity’ by EFSA (EFSA 2012b), the provisional HBGV was exceeded in 12 of 179 children up to 2-fold. Thus, the authors interpret this result as ’non-negligible exposure’ and suggest follow-up studies aimed to identify major sources of dietary CIT intake. HBM studies conducted in Asia and Africa have detected CIT and its metabolite DH-CIT in urine samples, and often at higher levels than those found in German and European population groups, and concurrently with the nephrotoxin OTA (Ali et al. 2016; Šarkanj et al. 2020).

Detection of a given mycotoxin biomarker in a biological sample is evidence of an exposure, but whether the levels present in urine or in other matrices will give rise to health concerns or not should be interpreted and communicated with some caution. An exposure which results in a HQ > 1 (due to exceedance of an HBGV or a MoE lower than recommended) is undesirable but it is not necessarily putting an individual at risk for adverse health effects. Tolerable intake levels should not be misunderstood as a demarcation for safe or unsafe exposures. It is worth noting that the derivation of HBGVs applies extrapolation factors (also termed ‘safety factors) to account for toxicodynamic and toxicokinetic differences between species and inter-individual variability in humans. Furthermore, a transient high exposure may elicit acute symptoms, e.g. vomiting in the case of DON, but no long-lasting effects when the exposure returns to low levels. This is different from exposure to carcinogenic and genotoxic mycotoxins which can have a long-term negative impact on human health. Of high interest is the exposure of neonates and infants in Sub-Saharan countries where mycotoxin contamination is prevalent. A recent study determined seven classes of mycotoxins in breast milk, complementary food and urine samples of infants aged 1–18 months (Ezekiel et al. 2022). The data obtained for exclusively and non-exclusively breast-fed infants provide insights into mycotoxin co-exposures in early life and highlight a transition to higher levels of exposure as complementary foods are introduced. Similar observations were made in the Gambia, where AF-Alb biomarkers at 4 months of age were only observed in infants that had transitioned to complementary foods (Turner et al. 2007). Thus, measures to mitigate mycotoxin exposure, also by promoting breast-feeding for at least 6 months are encouraged.

**Table 11:** Features for biomarkers and bio-measures of exposure in HBM studies

Mycotoxin Analytes	Matrix	Dose transferred/Fraction in matrix	Relevant time frame	Validated^§^	References
**Aflatoxin B**_1_					
AFM1	Urine	1–3%	24–48 h	Yes	Zhu et al. 1987
AFB1-Gua	Urine	0.2%	24–72 h	Yes	Groopman et al. 1993
AFB-Alb (AFB-Lys)	Serum/Plasma	1–3%	2–3 months	Yes	Gan et al. 1988
**Citrinin**					
CIT and DH-CIT	Urine	40%	1 day	Partial	Degen et al. 2018
CIT and DH-CIT	Serum/Plasma	n/e	1 day	n/e	
**Deoxynivalenol**					
DON and conjugates	Urine	65–75%	24–48 h	Yes	Turner et al. 2010b Warth et al. 2013a
DOM-1	Urine	< 5%	24–48^#^ h	No	Vidal et al. 2018a Turner et al. 2010a
**Fumonisins**					
FB1	Urine	0.5 (range 0.1–0.9)	24–48^#^ h	Yes	Riley et al. 2012
**Ochratoxin A**					
OTA	Serum/Plasma	n/e	several weeks	Partial	Studer-Rohr et al. 2000
OTA and conjugates	Urine	< 3%	n/e	Partial	Gilbert et al. 2001
OTα	Urine	n/e (variable)	n/e	No	Muñoz et al. 2014
**Tenuazonic acid**					
TeA	Urine	87–93%	≤ 24 h	No	Asam et al. 2013
	Urine	39 ± 22%	≤ 24 h	No	Visintin et al. 2023
**T-2 toxin**					
measured as HT-2 and conjugates	Urine	18.4%	12–24 h	No	McKeon et al. 2025
	Urine	17–20%			Sopel 2023
**Zearalenone**					
Sum of ZEN, α-ZEL & β-ZEL	Urine	10–20%	n/e	Partial	Metzler et al. 2010
ZEN and conjugates	Urine	7.9–13.2% in 1 day			Warth et al. 2013a

n/e: not established #: predicted but not established §: validated = biomarker instead of bio-measure - see Sect. Biomarkers for further details

### Toxicokinetic modelling

Toxicokinetic (TK) properties play a fundamental role in integrating HBGVs and HBM data in risk assessment. HBGVs are traditionally derived from point of departure (POD) determined in animal after oral exposure and, therefore, correspond to external exposure levels. Conversely, HBM data represent internal exposure values, reflecting the concentration of substances within the body. Integrating these two types of information requires a conversion step: either translating HBGVs into HBM-GVs or converting HBM data into Probable Daily Intake (PDI) by applying reverse dosimetry (Apel et al. 2020; Choi et al. 2017). This step is where TK and TK modelling become essential providing tools for bridging internal and external exposure metrics. Additionally, the popularity of TK modelling has thrived due to the possibility of quantitatively estimate TK and toxicodynamic (TD) variability used in risk assessment decision-making (Kreutz et al. 2024). One of the key advantages of TK modelling is its ability to quantify and account for human variability. Factors such as physiology, genetics, ontogenetics, exposomics, and even xenobiotic-microbiome interactions can all significantly influence the TK of a xenobiotic (Kreutz et al. 2024). Physiologically-based TK and population-based TK models account for these factors allowing for the estimation of a range of internal doses that more accurately reflect the diversity of population responses. These population-based approaches contrast with traditional risk assessment methods, which often rely on animal POD data and default tenfold uncertainty factors to account for interspecies and TK/TD differences (WHO/IPCS 2010). TK models have been used as tools for the estimation of chemical-specific adjustment factors (CSAFs) associated with the POD of both animal models and humans (Rezvanfar 2005). Driven by the need to estimate internal exposures where in vivo data generation is not possible, alternative testing strategies incorporating in vitro and in silico human specific models have recently gained interest. Physiologically-based TK frameworks are particularly well suited for *in vitro – in vivo* extrapolation (IVIVE). These types of population-based TK models allow for incorporation of in vitro data and in silico predictions to derive human steady-state blood concentrations equivalents (Chang et al. 2022). Consideration of variability of these data allows for extrapolation of a range of in vivo equivalent doses across individuals rather than a single value, which more accurately reflects the distribution of population responses that occur from exposure to a chemical (Wetmore et al. 2014; Visintin et al. 2025). Traditionally, population-based TK modelling approaches are conducted on one chemical at a time, but new approaches employing high-throughput in vitro testing and computational modelling to predict internal exposures - including quantitative estimates of population variability - have been employed on environmental xenobiotics (Jamei et al. 2009; Rotroff et al. 2010; Bessems et al. 2014; Pearce et al. 2017). When conducting an observational study on a study population interviewed using a 24-hour dietary recall or an FFQ, population-based TK modelling provides the opportunity to derive population TK properties for the xenobiotics detected, based on the estimated external exposure and HBM data (Amzal et al. 2009). As mentioned, carefully designing the study and FFQ can significantly enhance the interpretation of HBM data by providing complementary dietary exposure information (Heyndrickx et al. 2015). However, the applicability of this approach is often hindered by the low concentration and sporadic occurrence of mycotoxins, which results in a loss of statistical power. This challenge must be mitigated by recruiting a large number of participants, a consideration that should be addressed during the study’s design phase, taking into account also ethical considerations.

In the field of mycotoxicology, despite the existence of hundreds of known mycotoxins, TK models have been developed for a limited number of mycotoxins in humans, including DON, AFB1, OTA, and TeA. Additionally, IVIVE was applied to selected mycotoxins for the derivation of the kinetics of EnB and EnB1 from cellular models by Fæste et al. (2011) and Ivanova et al. (2019), respectively. The primary objectives of these models include predicting the TK properties from in vivo or in vitro data, deriving benchmark doses, and calculating CSAFs (Fæste et al. 2018; Gilbert-Sandoval et al. 2020; Lootens et al. 2023; Mengelers et al. 2019; Su et al. 2024; Visintin et al. 2025). TK information for mycotoxins is often scarce and fragmented. Consequently, TK modelling still holds significant potential to make impactful contributions in the field, supported by the growing availability of diverse in silico resources accessible to the scientific community (Madden et al. 2019). For instance, the freeware IndusChemFate model, developed for multiple exposure routes, was fitted for oral exposure to DON (Notenboom et al. 2023), enabling the possibility to simulate aggregate exposure in occupational contexts (Viegas et al. 2018).

## Conclusion and recommendation for future research

Human biomonitoring based on the analysis of blood, urine or breast milk samples provides a powerful approach for exposure assessment of mycotoxins and has been widely used in the past. However, there are several major challenges and pitfalls to be considered to achieve reliable and standardized data for mycotoxin risk assessment. These challenges and pitfalls have been extensively discussed in this review in the sections above covering the topics: Biomarkers, Study design and dietary intake records, Matrices, sampling, clean-up and mass spectrometric analysis as well as Recent trends and challenges in interpretation of human biomonitoring data. In the following section the current status of the above discussed topics relevant for mycotoxin HBM studies are summarized as bullet points marked with #, major pitfalls and recommendations for future HBM studies are marked with →:

- **Biomarker**.
  # Just a few biomarkers of exposure for HBM of mycotoxins have been fully validated.
  → Studies, preferably in humans, of the natural exposure to dietary mycotoxins should be performed to validate biomarkers where gaps exist to create an intake versus bio-measure ratio.
  → Human data on mycotoxin toxicokinetics are missing for many exposures. Such data would allow improved reverse dosimetry estimates and may inform/guide optimal sampling strategies.
  → Need for research on more long-term biomarker (e.g. other protein adducts such as albumin, haemoglobin or adducts with other macromolecules).
  → The term biomarker should be used only for compounds which are fully validated otherwise the term bio-measure is recommended.
- **Cohorts/Study design and FFQ**.
  # Different cohort study designs are available and have been used in the past for HBM of mycotoxins.
  → Use prospective cohort designs for better temporal causality and enable linking long-term exposure to chronic outcomes and capture intra-individual variability over time.
  → Use of validated FFQ including specific questions tailored to mycotoxin research, or food diaries.
  → Record frequency and portion of food intake and document seasonal food availability and consumption patterns.
  → Include other omics techniques to discover novel biological changes and endpoints.
- **Matrices and Sampling**.
  # Sampling techniques for common matrices such as blood, urine or breast milk have been well established.
  → As gold standard the use of 24-hour urine sampling should be preferred over spot urine samples. For spot urine, it is recommended to record the time since the last meal, especially for compounds with a short half-life.
  → Micro-sampling techniques should be optimized to lower blood sample volumes as this is often a limiting factor in human studies.
  → Caution is needed when comparing mycotoxin levels in different hematic matrices such as plasma, serum, whole blood and/or VAMS as conversion factors are not established for all mycotoxins.
  → When performing blood withdrawals, it is important to record the time since the last meal, as fasting significantly influences the measured mycotoxin biomarkers.
  → Stability of mycotoxin biomarkers in different matrices during collection, transport and over time in storage including different temperatures (−20 and − 80 °C) should be assessed.
- **Analysis**.
  # Several multi-mycotoxin methods have been established and validated for all common matrices.
  → Need of more and larger interlaboratory studies including cross-validation of analytical methods.
  → Multi-mycotoxin methods should be optimized for high-throughput analysis, e.g. by the use of online-clean-up-techniques.
  → Multi-mycotoxin methods should be further optimized to include missing biomarkers and to lower LOD/LOQ in order to avoid left censored data.
  → Rapid, low-cost assays (including immunoassay-based tests) should be developed for applications in low-resource settings.
  → Missing mycotoxin standards, especially isotope-labelled standards, as well as reference material for HBM such as AFB1-Lys should be provided by chemical synthesis and made commercially available.
- **Biomarker Interpretation/Exposure Evaluation/Risk Assessment/Toxicokinetics**.
  # HBM represents a powerful approach to assess human exposure (from all routes and sources). However, biomarkers of mycotoxin exposure are critical for the interpretation of the exposure-health relationships reported in human observational studies.
  → For numerous mycotoxins toxicokinetic data in humans including excretion rates and characterization of major metabolites are needed to guide HBM studies.
  → Health-based guidance values (HBGVs) are missing for most mycotoxins and are needed for valid HBM interpretation.
  → HBGVs should ideally be derived from human epidemiological data or be determined based on in vivo animal studies. In the future, alternative approaches such as in vitro-in vivo extrapolation (IVIVE) or organ-on-chip (OoC) and integrated toxicokinetic modelling might also be useful.
  → Reliable correlation analysis of validated biomarkers with food intake date, including minor components such as spices, are strongly recommended for all HBM studies.
  → For statistical power large-cohort studies should be performed to estimate exposure, however both the variance in the biomarker estimates and ethical considerations should be taken into account.
  → Physiological samples are valuable and in future studies, besides single mycotoxins and mixtures of mycotoxins, exposome studies should be performed more intensively to evaluate potential health risks of environmental exposure more generally.

## Supplementary Information

Below is the link to the electronic supplementary material.

ESM 1 (DOCX 619 KB)

## Data Availability

No datasets were generated or analysed during the current study.
