# Supplementary material for: Human biomonitoring of mycotoxins: key challenges and future directions
Source: Mycotoxin Res. 2025 Dec 23;42(1):13. doi: 10.1007/s12550-025-00612-2 (PMC12727801; doi:10.1007/s12550-025-00612-2)
Supplement: Supplementary file 1 — (DOCX 619 KB) [file 12550_2025_612_MOESM1_ESM.docx]

**Human Biomonitoring of Mycotoxins: Key Challenges and Future Directions**

Benedikt Cramer^1^, Lia Visintin^2,3^, Elias Maris^2,4^, Michael Kuhn^1^, Gisela H. Degen^5^, Paul C. Turner^6^, Hans-Ulrich Humpf^1*^, Sarah De Saeger^2,7*^

^1^ Institute of Food Chemistry, University of Münster, Corrensstraße 45, 48149, Münster, Germany.

^2^ Department of Bioanalysis, Centre of Excellence in Mycotoxicology and Public Health, Faculty of Pharmaceutical Sciences, Ghent University, 9000 Ghent, Belgium.

^3^ Department of Diagnostic Sciences, Doping Control Laboratory, Faculty of Medicine and Health Sciences, Ghent University, 9000 Ghent, Belgium

^4^ Department of Microbiology and Immunology, Laboratory of Molecular Bacteriology, Rega Institute, KU Leuven, 3000 Leuven, Belgium.

^5^ Leibniz Research Centre for Working Environment and Human Factors (IfADo), Ardeystraße 67, 44139, Dortmund, Germany.

^6^ Department of Global, Environmental and Occupational Health, School of Public Health, University of Maryland, College Park, Maryland, USA.

^7^ Department of Biotechnology and Food Technology, Faculty of Science, University of Johannesburg, P.O. Box 17011, Doornfontein Campus, Johannesburg, South Africa.

Corresponding authors: [sarah.desaeger@ugent.be](mailto:sarah.desaeger@ugent.be) (SDS), [humpf@uni-muenster.de](mailto:humpf@uni-muenster.de) (HUH)

**Aflatoxins**

***Alternaria*-Toxins α**

**Ochratoxins**

**Citrinin**

**Deoxynivalenol**

**Other Trichothecenes**

**Beauvericin and Enniatins**

**Fumonisins**

**Ergotalkaloids**

**Zearalenone**

**Other mycotoxins**
